# Stable isotopic investigation of the feeding ecology of wild Bornean orangutans

**DOI:** 10.1002/ajpa.24598

**Published:** 2022-08-10

**Authors:** Takumi Tsutaya, Anna Wong, Peter T. Malim, Henry Bernard, Nanako O. Ogawa, Naohiko Ohkouchi, Shun Hongo, Tomoyuki Tajima, Tomoko Kanamori, Noko Kuze

**Affiliations:** ^1^ Department of Evolutionary Studies of Biosystems, Research Center for Integrative Evolutionary Science the Graduate University for Advanced Studies Hayama Kanagawa Japan; ^2^ Biogeochemistry Research Center, Research Institute for Marine Resources Utilization Japan Agency for Marine‐Earth Science and Technology Yokosuka Kanagawa Japan; ^3^ Japan Orangutan Research Center Tokyo Japan; ^4^ Faculty of Tropical Forestry Universiti Malaysia Sabah Kota Kinabalu Sabah Malaysia; ^5^ Sabah Wildlife Department Kota Kinabalu Sabah Malaysia; ^6^ Institute for Tropical Biology and Conservation Universiti Malaysia Sabah Kota Kinabalu Sabah Malaysia; ^7^ The Center for African Area Studies Kyoto University Kyoto Japan; ^8^ Unit of Synergetic Studies for Space Kyoto University Kyoto Japan; ^9^ Primate Research Institute Kyoto University Inuyama Aichi Japan; ^10^ Department of Anthropology National Museum of Nature and Science Ibaraki Japan

**Keywords:** carbon and nitrogen, diet, feces, plants, stable isotope analysis

## Abstract

**Objectives:**

We applied stable carbon and nitrogen isotope analyses to wild Bornean orangutans (*Pongo pygmaeus morio*) to investigate the feeding ecology of wild orangutans. Compared with African great ape species, orangutans are adapted to environments with chronic lower nutrition. But the usefulness of stable isotope analysis in the study of wild orangutan feeding ecology has not been fully explored.

**Methods:**

The study site was a primary lowland dipterocarp forest in the Danum Valley, Sabah, Malaysia. A total of 164 plant and 94 fecal samples collected across 18 months were analyzed.

**Results:**

Carbon and nitrogen stable isotope ratios of plant food samples do not systematically vary by plant parts (i.e., bark, fruits, and leaves). Elemental composition and stable isotope ratios of orangutan feces do not systematically vary by orangutans' sex and age classes, although fecal stable isotope ratios showed seasonal fluctuations. No isotopic contribution of breast milk was found in fecal samples collected from individuals at 2.7–6.5 years of age.

**Conclusions:**

This study revealed key characteristics of the stable isotope ecology of wild orangutans living in a primary lowland forest. Although there was little isotopic variation among plant foods and orangutan individuals, seasonal fluctuations in baseline isotope ratios or orangutans' diet were found in Danum valley.

## Graphical abstract

Flanged male orangutan eating bark.

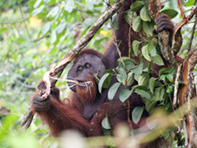

## 1 INTRODUCTION

Orangutans are the only existing great ape species that live outside of Africa. Orangutans diverged from the human ancestral clade 12–16 million years ago, and ~ 97.4% of their genomic nucleotide sequence is also found in humans (Locke et al., 2011). Today, three orangutan species exist on Borneo (*Pongo pygmaeus*) and Sumatra (*P. abelii* and *P. tapanuliensis*) (Nater et al., 2017). Fossil records show that orangutans were distributed throughout Southeast Asia during the Pleistocene (Ibrahim et al., 2013). After this time, distribution was confined to Borneo and Sumatra. All three existing orangutan species are categorized as Critically Endangered on the IUCN Red List (Ancrenaz et al., 2016; Nowak et al., 2017; Singleton et al., 2017), and an understanding of their ecology is urgently needed for more efficient conservation (Meijaard et al., 2012; Spehar et al., 2018).

The diet of wild orangutans consists mostly of plant materials, and feeding behaviors show flexibility in diverse forest environments (Russon et al., 2009). Primary food sources are fruits in forests, and the proportion of fallback foods, such as bark, increases during times of lower productivity (Kanamori et al., 2010). Orangutans are adapted to environments where there are long periods of low availability of preferred foods with high caloric values (Pontzer et al., 2010), and the amount of fruit consumed greatly increases during the mast fruiting period (Knott, 1998). Also, orangutans show ecological resilience in anthropogenically degraded landscapes, such as plantations and timber concessions (Ancrenaz et al., 2010, 2015; Campbell‐Smith et al., 2011a, 2011b; Meijaard, Welsh, et al., 2010). Changes in orangutan diet in such degraded landscapes are shown or suggested in some studies (Ancrenaz et al., 2015; Campbell‐Smith et al., 2011a).

This study investigates stable carbon and nitrogen isotope ratios (δ^13^C and δ^15^N, respectively) of orangutans and their foods to characterize the feeding ecology of a wild orangutan population living in a primary forest. Stable isotope analysis has been used to investigate the diet of extant primate species and to reconstruct the paleoenvironment using fossil materials (Crowley, 2012; Kohn & Cerling, 2002; Sandberg et al., 2012). Many isotope studies are available for African great ape species, but few isotope studies have been reported in wild orangutans. Stable carbon and nitrogen isotope analyses have been used for investigating the seasonality of diet, inter‐ and intra‐species differences in diet and habitat use in chimpanzees, bonobos, and gorillas (e.g., Blumenthal et al., 2012; Fahy et al., 2013; Loudon et al., 2016, 2019; Oelze et al., 2011, 2014, 2016, 2020). Conversely, no stable isotope studies are available for investigating the feeding ecology of wild orangutans, as far as the authors are aware. To date, stable isotope analyses have been used only to investigate nutritional stress in Bornean orangutans (Vogel, Crowley, et al., 2012; Vogel, Knott, et al., 2012), where the relationship between δ^15^N values of urine and nutritional stress was investigated. Therefore, the utility of stable isotope analysis in ecological studies of orangutans is unclear and requires detailed investigation.

### 1.1 Stable isotope analysis in primate ecology

Stable carbon and nitrogen isotopes naturally occur and can be used in primatology and human evolution studies to assess habitat, food sources, and breastfeeding and weaning patterns (Crowley, 2012; Kohn & Cerling, 2002; Sandberg et al., 2012; Tsutaya & Yoneda, 2015). Stable carbon and nitrogen isotope ratios of biological compounds vary systematically depending on ecological and physiological processes, and isotopic signatures are recorded in plant and primate body tissues. Thus, ecological/physiological processes can be evaluated by measuring isotope ratios in primate tissues and their dietary sources, as described below.

Stable carbon isotope ratios in plant parts are primarily affected by ecological conditions during the uptake and assimilation of CO_2_ (Dawson et al., 2002). A large δ^13^C difference is found among plants with different photosynthetic systems. For example, C_3_ plants exhibit ~15‰ lower δ^13^C values compared with C_4_ plants (O'Leary, 1988; Smith & Epstein, 1971). Light and water conditions during photosynthesis further affect δ^13^C values: lower light intensity typically decreases carboxylation and δ^13^C values and lower water availability or lower humidity typically induces stoma closure, leading to decreased CO_2_ concentration in cells and higher δ^13^C values (Heaton, 1999). These two factors result in δ^13^C values in plants living in closed habitats with lower light intensity and higher humidity (e.g., dense forest) to be up to ~5‰ lower than those in open habitats (e.g., grassland and large gap) (van der Merwe & Medina, 1989; van der Merwe & Medina, 1991). Furthermore, soil respiration releases ^13^C‐depleted CO_2_ from the ground surface, generating a vertical gradient of δ^13^C values inside forest canopies, and δ^13^C values of CO_2_ in plants tend to increase by ~5‰ from the ground to the canopy (“canopy effect”) (Lowry et al., 2021; Medina et al., 1991; van der Merwe & Medina, 1991). Finally, the change in baseline δ^13^C in atmospheric CO_2_ results in long‐term differences in plant δ^13^C values (Heaton, 1999). Fossil fuel combustion has added ^13^C‐depleted CO_2_ to the atmosphere, and a typical modern plant shows ~1.5‰ decreased δ^13^C value compared with plants that grew before the industrial revolution (Friedli et al., 1986; Suess, 1955), which is relevant when comparing extant and extinct species.

Stable nitrogen isotopes are used for investigating trophic relationships in ecosystems. δ^15^N values in plants are primarily determined by the source of nitrogen compounds, although various other factors, such as the availability of source nitrogen and nitrogen demand, also affect plant δ^15^N (Dawson et al., 2002; Evans, 2001; Szpak, 2014). δ^15^N values of nitrogen compounds change from the baseline source value after assimilation into a food web. A 3–4‰ stepwise enrichment in relationships is seen between prey and predator, and δ^15^N values are typically higher at higher trophic levels (Minagawa & Wada, 1984; Schoeninger & DeNiro, 1984). Assimilation of nitrogen from organic fertilizers (e.g., animal manure and human food wastes) with relatively higher δ^15^N values compared with natural inorganic nitrogen (e.g., nitrate and ammonium) increases δ^15^N (Bogaard et al., 2013; Szpak, 2014).

Different plant parts usually contain different compounds derived from various nutritional pathways, and these metabolic differences result in variations in carbon and nitrogen elemental concentrations (weight percent of carbon and nitrogen in a given sample [%C and %N]) and stable isotope ratios. For example, lignin and cellulose are primary structural compounds of plant tissues, and their proportion is generally greater in hard plant parts, such as bark. The δ^13^C in bark is higher because δ^13^C in cellulose is 1–2‰ greater and in lignin is 2–6‰ greater than in the whole C_3_ plant (Benner et al., 1987; Loader et al., 2003). Non‐photosynthetic tissues, such as fruits, generally show greater δ^13^C values compared with leaves in C_3_ plants, though the mechanism is unclear (Cernusak et al., 2009).

Habitats and food sources of various extant and fossil primate species have been reconstructed utilizing the above principles of isotope geochemistry and biological processes (Crowley, 2012; Sandberg et al., 2012). Carbon and nitrogen stable isotope ratios in primate tissues directly reflect ratios in plant sources with up to ~4‰ fractionation (e.g., Macharia et al., 2014; Tsutaya et al., 2017). The contribution of food sources produces distinct isotope signatures in animal tissues. These signatures reflect ecological conditions of subject species' habitat use (e.g., openness and vertical height). Thus, habitat can be examined using δ^13^C and δ^15^N values of primate tissues (e.g., Schoeninger, 2014; Sponheimer et al., 2009; White et al., 2009). The anthropogenic impact on the diet of extant wild nonhuman primate species has also been studied using stable isotope methodologies (Loudon et al., 2014; Schurr et al., 2012).

Stable isotope analysis has also been used to reconstruct breastfeeding and weaning patterns in primates (summarized in Tsutaya & Yoneda, 2015). Consumption of breast milk increases δ^15^N values in infants through bioenrichment between mother and infant (Fuller et al., 2006). δ^15^N values of infant body tissues typically increase compared with maternal tissues by ~2–3‰ during the exclusive breastfeeding period. δ^15^N values then start to decrease after the introduction of solid foods and become similar to maternal values after weaning (Bădescu et al., 2017, 2022; Fahy et al., 2014; Reitsema, 2012; Reitsema et al., 2015). Further, elemental nitrogen concentrations of breast milk are higher than in most plant foods, and fecal nitrogen concentration shows a similar pattern of increase and decrease in parallel with breastfeeding and weaning (Bădescu et al., 2017, 2022; Reitsema, 2012).

### 1.2 Hypotheses tested in this study

In this study, carbon and nitrogen stable isotopes were measured in plant food and fecal samples of wild Bornean orangutans in the Danum valley conservation area (DVCA: Sabah, Malaysia) to characterize their feeding ecology. We tested the following five hypotheses to validate the utility of stable isotope analysis in studying the feeding ecology of wild orangutans.

1. Isotopic variation among plant parts: Different types of food plant parts (e.g., fruits and leaves) will show significantly different stable isotope ratios as can be seen in African and Asian tropical forests (e.g., Blumenthal et al., 2012, 2016; Oelze et al., 2014; Roberts et al., 2017). Such a difference is useful to estimate the contribution of different food sources in the diet of primate individuals.
2. Individual variation in fecal isotope ratios: Stable isotope ratios of feces will differ among individuals of different ages and sex because of their dietary variations. The dietary breadth and macronutrient intake can differ in orangutan individuals of different ages and sex (Schuppli et al., 2016, 2021; Vogel et al., 2017; but see Wich et al., 2006), and subtle individual dietary differences are reported in orangutans in DVCA (Kanamori et al., 2010). Such differences may be detectable by using stable isotope analysis.
3. Seasonal variation in fecal isotope ratios: Stable isotope ratios of feces will change with seasonality as a reflection of changes in diet with seasonality. Lowland dipterocarp forests in Borneo show intra‐annual and supra‐annual fluctuations in the amount of available food sources (Kanamori et al., 2010; Knott, 1998). If the stable isotope ratios of these food sources differ, such fluctuations may be seen in the stable isotope ratios of orangutan feces.
4. Isotopic effect of breastfeeding: Feces of breastfed infants and juveniles will show greater δ^15^N values compared with those of mothers because of the effect of breastfeeding (Bădescu et al., 2017, 2022; Fahy et al., 2014; Fuller et al., 2006; Reitsema, 2012; Reitsema et al., 2015; Tsutaya & Yoneda, 2015). Such a difference is useful to estimate breastfeeding and weaning patterns in orangutans.
5. Variation in elemental concentrations of feces: The elemental concentration of feces will differ by age, sex, season, and breast milk consumption. Because the elemental concentration of feces is affected by that of consumed foods (Bădescu et al., 2017; Reitsema, 2012), consumption of food sources with different elemental concentrations may be detectable by analyzing the feces of orangutans.

Furthermore, we evaluate the utility of applying stable isotopic data from wild extant orangutan populations to improve the accuracy of paleoenvironmental reconstruction. Past ecological settings, especially the type of vegetation, can be reconstructed from bioapatite δ^13^C values of herbivore hard tissues, such as teeth and bones, because δ^13^C values in herbivore tissues systematically vary with the dietary proportion of C_4_ plants and openness of habitat (Schoeninger, 2014; Sponheimer et al., 2009; White et al., 2009). Chronological changes in bioapatite δ^13^C values from Southeast Asian mammals suggest that some parts of forestland during the Early Pleistocene changed to savannah by the Middle Pleistocene, and this change led to habitat contraction for herbivores, such as orangutans (Louys & Roberts, 2020; Wurster et al., 2019). Fossil records suggest that the habitat of orangutans decreased substantially after the Late Pleistocene (Ibrahim et al., 2013), possibly because of these changes in vegetation. Moreover, extant orangutan populations might have been driven into less accessible forests for humans, such as peat swamp and upland forests, to avoid pressures from anthropogenic activities (Ancrenaz et al., 2010; Meijaard et al., 2021; Meijaard, Albar, et al., 2010). However, relating fossil δ^13^C values to certain environmental conditions is difficult, and fossil δ^13^C values provide only a rough indication of past environments. Stable isotope data for wild orangutans and their food sources from well‐contextualized modern forests can be used as an accurate proxy to interpret δ^13^C of ancient species, as has been shown in chimpanzees (Nelson, 2013). Using detailed information on relationships among environmental conditions and δ^13^C values of plants and consumers as a modern proxy, more accurate paleoenvironmental reconstructions from fossil specimens are possible.

## 2 MATERIALS AND METHODS

### 2.1 Ethical statements

Permissions for conducting research and collecting samples at the DVCA and for the export of fecal and plant samples to Japan were granted by the Sabah state government. This study was conducted in compliance with the animal care regulations and laws of the state government of Sabah, Malaysia (Access Licenses JKM/MBS.1000‐2/2 JLD.3 (65) and JKM/MBS.1000‐2/2 JLD.4 (87)). Export of fecal and plant samples to Japan was granted by the Sabah state government (Transfer License JKM/MBS.1000‐2/3 JLD.3 (46)).

### 2.2 Study site and sample collection

DVCA is ~438 km^2^ located in northeast Borneo, Sabah, Malaysia. The area is predominantly lowland dipterocarp forest interspersed with some lower‐hill dipterocarp forest and is characterized by comparatively low fruit availability among rain forests in the world (Hanya et al., 2011). This area was established as a forest reserve by the Sabah state government in 1996 and is managed by the Yayasan Sabah Group (the Sabah Foundation) for conservation, research, education, and tourism. Our study site (5°01′17′′ N, 117°44′50′′ E, 231–384 m a.s.l.) is a primary forest of ~2 km^2^ area surrounding the Borneo Rainforest Lodge (BRL). The lodge caters to tourists and is managed by the Yayasan Sabah Group. The mean ± standard deviation (SD)  for monthly rainfall recorded at Danum Valley Field Research Center (DVFC) of the DVCA (~15 km south from BRL) during the study period was 242.6 ± 108.8 mm (min, 78.2 mm; max, 464.9 mm).^1^ Rainfall tended to be higher during October–March and May and lower during April and June–September (Kanamori et al., 2017).

Long‐term research on wild orangutans (*P. pygmaeus morio*) in the DVCA has been ongoing since 2004, and > 50 orangutan individuals have been identified and monitored (Kanamori et al., 2010, 2017; Kuze et al., 2011; Mendonça et al., 2016, 2017). The mean orangutan density in this area is calculated as 1.3 individuals/km^2^ from nest census data, and the density increases during mast and peak fruiting seasons (Kanamori et al., 2017). Age classes of orangutans are defined as infant (0–2 years old), juvenile (3–7 years old), adolescent (8–15 years old), and adult (individual aged ≥16 years old) (Kuze et al., 2005). Adult male individuals were further classified as flanged or unflanged by the presence or absence of the flange, a cheek pad that develops in dominant adult male orangutans. All adult female individuals analyzed in this study had dependent offspring during the study period. We observed that all adolescent female individuals were nulliparous until the end of the study period (Table S1).

Fecal samples were mostly picked up by hand with disposable plastic gloves and collected inside a plastic bag by researchers and local research assistants during routine behavioral observation of orangutan individuals for 18 months from September 2015 to February 2017. A total of 94 orangutan fecal samples from 24 individuals were collected and analyzed (Table S2). Due to the low number of observed infant individuals (*n* ≤ 3) during the study period in BRL, as well as the difficulty in the collection of tiny infant feces that dropped from the higher canopy, only one fecal sample from an infant was available for this study. Two samples with outlier values were excluded from the data set (Supplementary Text 1.1 in Data S1). The stable isotope ratios of fecal samples collected from the same individual during the same month were averaged prior to statistical tests and visualization (except for linear mixed‐effects models: LMMs) to avoid pseudo‐replication, resulting in 66 data points generated from fecal samples.

Plant samples were obtained from the same parts of the same plant individual at the same tree height and same developmental stage (e.g., mature or unripe fruits) to avoid bias resulting from vertical stratification and difference in developmental stages (Blumenthal et al., 2016; Roberts et al., 2017). During the feeding bout of a focal individual, a research assistant collected portions of the same food source dropped by the focal individual. Therefore, only the food samples eaten by the focal individual at that moment were used in this study. Food samples collected during the two rainfall periods (Period 1: January and February 2016, Period 2: July, August, and October 2016) were used as representatives for periods with relatively lower and higher amounts of rainfall during a year, respectively. A total of 164 samples from at least 54 species were analyzed (Table S3). Ten samples with outlier values and four samples lacking taxonomic information were excluded from the data set (Supplementary Text 1.1 in Data S1). Stable isotope ratios of plant samples collected from the same species and parts during the same season were averaged prior to statistical tests and visualization, except for LMMs, to avoid pseudo‐replication, and 72 data points were generated from 61 combinations of species and parts. The taxonomy of plant species was confirmed by an experienced botanist (Mike Bernadus) in DVFC.

Fecal and food samples were stored at −18°C inside a freezer in the research station of BRL within 12 h after sampling. Then, frozen plant food samples were completely dried at 60°C by placing them inside an electric oven for more than 18 h. Frozen fecal and dried food samples were exported from Malaysia to Japan for the measurement of stable isotope ratios.

### 2.3 Stable isotope analysis

Exported samples were processed for stable isotope analysis. All samples were freeze‐dried before processing. Plant food samples were crushed into a fine powder using a stainless mortar and pestle. Bark samples were cut into tiny pieces using a pair of scissors. Applying the method described by Tsutaya et al. (2017) and Tsutaya, Ogawa, et al. (2021), we crushed fecal samples mildly, sieved them with 0.5‐mm mesh, and used the dropped powder for analysis. The freeze‐dried samples of ~0.03–0.04 mg were placed in pre‐cleaned tin capsules. A separate analysis used ~0.1 mg subsamples to determine δ^15^N value for food materials with low nitrogen content, such as bark.

Carbon and nitrogen stable isotope ratios were measured using a modified elemental analyzer/isotope mass spectrometer at the Japan Agency for Marine‐Earth Science and Technology. Instrumentation consisted of an elemental analyzer (Flash EA 1112, ThermoFinnigan, Bremen, Germany) coupled to an isotope ratio mass spectrometer (Delta plus XP, ThermoFinnigan, Bremen, Germany) through a continuous flow interface (ConFloIII, ThermoFinnigan, Bremen, Germany) with modification specifically made to improve the sensitivity of the analysis (Ogawa et al., 2010). Carbon and nitrogen isotope ratios were expressed in δ notation relative to standards, δ^13^C (‰) and δ^15^N (‰), with Vienna Pee Dee Belemnite (VPDB) and atmospheric nitrogen (AIR) as the standard for carbon and nitrogen, respectively. Isotope ratios were calibrated using three laboratory standards: L‐tyrosine (δ^13^C = −20.83 ± 0.10‰, δ^15^N = 8.74 ± 0.04‰), L‐proline (δ^13^C = −10.27 ± 0.04‰, δ^15^N = 13.51 ± 0.02‰), and DL‐alanine (δ^13^C = −25.36 ± 0.08‰, δ^15^N = −2.89 ± 0.04‰). These isotope compositions were calibrated with authentic standards (Tayasu et al., 2011). The analytical errors (1σ) determined by the repeated measurements of L‐tyrosine (BG‐T) were typically ±0.34‰ and ± 0.29‰ for δ^13^C and δ^15^N, respectively.

### 2.4 Data analyses

All data analyses used R software, version 4.0.4 (R Core Team, 2021). The R package “rKIN” was used to visualize data distributions (Eckrich et al., 2020). Statistical significance was set at α ≤ 0.05.

The R package “lme4” was used to construct LMMs to investigate the variability of carbon and nitrogen elemental concentrations and stable isotope ratios of plant foods and orangutan feces, as response variables (Bates et al., 2015). To test isotopic variation among plant parts (Hypothesis 1), we constructed the model considering parts (bark, fruit, or leaves) and rainfall period (1 or 2) as a fixed effect and the combination of species and parts (1|species_part) as a random effect for the analysis of plant stable isotope ratios. To test individual and seasonal varition in fecal isotope ratios (Hypotheses 2 and 3), we constructed the model considering five fixed effects of sex (female or male), age class (infant/juvenile, adolescent, or adult), the sex–age interaction, and sine and cosine of the sampling date within the year (transformed into a periodic form [date × 2 × π/365]) and a random intercept effect of individual ID for the analysis of fecal stable isotope ratios. To test the variation in elemental concentrations of feces (Hypothesis 5), we constructed a model that has the same linear‐explanatory structure as that for Hypotheses 2 and 3 but considers fecal elemental concentrations as the response variable. Effects of explanatory variables were tested using the Student's *t* test.

Comparative bioapatite δ^13^C values of fossil orangutans, compiled by Louys and Roberts (2020), were used to contextualize the results of this study for palaeoecological reconstruction. The Louys and Roberts (2020) data set consists of previously reported data (Bacon et al., 2018; Bocherens et al., 2017; Pushkina et al., 2010; Qu et al., 2014) and newly obtained data from paleontological and historical orangutan fossil specimens from the Pleistocene and Holocene in Southeast/East Asia. In this study, carbon isotope offset values of diet–bioapatite and diet–feces were set as −11.8‰ (Malone et al., 2021) and − 1.84‰ (Tsutaya, Ogawa, et al., 2021), respectively, adopting ratios reported in previous literature. The Suess effect was considered when comparing fecal δ^13^C with fossil δ^13^C, and 1.5‰ was subtracted from ratios of fossil specimens (Friedli et al., 1986).

## 3 RESULTS

### 3.1 Plant foods

**FIGURE 1:**
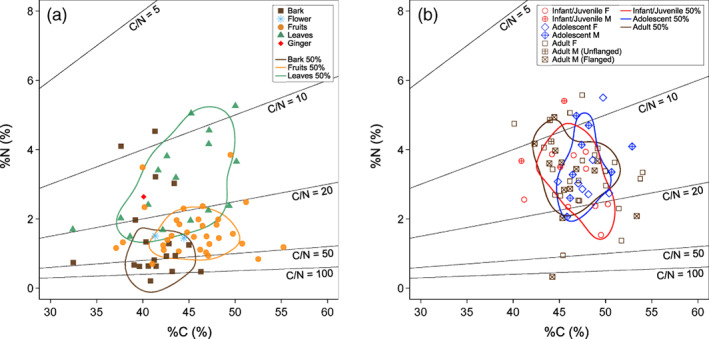
Elemental carbon and nitrogen concentration of (a) dietary plant food and (b) fecal samples from wild orangutans in Danum valley conservation area. Enclosed areas represent 50% kernel density ranges (Eckrich et al., 2020). Solid black lines represent points corresponding to specific elemental C/N ratios

**FIGURE 2:**
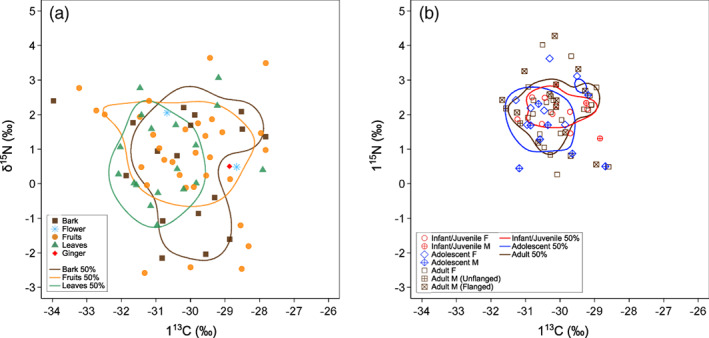
Carbon and nitrogen stable isotope ratios of (a) dietary plant food and (b) fecal samples from wild orangutans in Danum valley conservation area. Enclosed areas represent 50% kernel density ranges (Eckrich et al., 2020)

Mean δ^13^C and δ^15^N values of all plant samples in different combinations of species, parts, and rainfall period were − 30.3 ± 1.4‰ (from −34.5‰ to −27.8‰ in the full range) and 0.7 ± 1.4‰ (from −2.6‰ to 3.6‰ in the full range), respectively (*n* = 71) (Table 1; Figures 1 and 2). The δ^13^C values in measured samples were entirely within the range of C_3_ plants (O'Leary, 1988; Smith & Epstein, 1971). Isotope ratios of plant food samples in the DVCA are consistent with typical values reported in plants from the canopy of Bornean lowland rainforests (Hyodo et al., 2010; Tanaka‐Oda et al., 2016; Supplementary Text 1.2 in Data S1).

**TABLE 1:** Summary of elemental contents and stable isotope ratios of dietary plant food samples

	%C		%N		δ^13^C		δ^15^N		
	Mean	SD	Mean	SD	Mean	SD	Mean	SD	*n*
Bark	41.15	2.98	1.47	1.29	−30.41	1.76	0.48	1.54	19
Flower	42.89	2.20	1.48	0.04	−29.67	1.43	1.27	1.11	2
Fruits	45.60	4.10	1.66	0.70	−30.09	1.39	0.77	1.58	32
Leaves	44.28	4.91	3.06	1.25	−30.63	1.11	0.80	1.21	18
Other	40.10	−	2.64	−	−28.87	−	0.50	−	1

No significant effect was seen in the plant part and rainfall period as explanatory variables for the non‐averaged plant stable isotope ratios (Table 2). This finding is consistent with the non‐systematic difference in %C, %N, δ^13^C, and δ^15^N values among the different parts of the same plant species and between the same plant species + parts collected in different rainfall periods (Supplementary Text 1.3 in Data S1; Figures S1 and S2; Tables S4 and S5).

**TABLE 2:** Explanatory variables and intercept in linear mixed models for plant stable isotope ratios

Response variables	Explanatory variables		Effect	SE	t	*p*‐value
δ^13^C	Fixed effect	**Intercept**	**−30.58**	**0.38**	**−79.79**	**<0.001**
		Tissue: Flower	0.38	1.37	0.28	0.783
		Tissue: Fruit	0.36	0.46	0.80	0.429
		Tissue: Leaves	−0.46	0.53	−0.87	0.387
		Tissue: Other	1.71	1.47	1.16	0.251
		Period: 1	0.57	0.30	1.92	0.057
	Random effect	SD of Species × Tissue	1.19	1.09	–	–
δ^15^N	Fixed effect	Intercept	0.03	0.40	0.08	0.939
		Tissue: Flower	1.50	1.31	1.14	0.258
		Tissue: Fruit	0.88	0.46	1.93	0.060
		Tissue: Leaves	0.82	0.52	1.56	0.125
		Tissue: Other	0.47	1.54	0.31	0.760
		Period: 1	0.01	0.38	0.02	0.986
	Random effect	*SD* of Species × Tissue	0.35	0.59	–	–

*Note*: Significant fixed effects are shown in bold.

### 3.2 Fecal samples

Mean δ^13^C and δ^15^N values of fecal samples in different combinations for individual and month were − 30.2 ± 0.7‰ (from −31.7‰ to −28.6‰, *n* = 65) and 2.1 ± 0.8‰ (from 0.8‰ to 2.1‰ in the full range, *n* = 66) (Table 3; Figures 1 and 2).

**TABLE 3:** Summary of the elemental contents and stable isotope ratios of fecal samples from wild orangutans in Danum valley conservation area

		%C		%N		δ^13^C		δ^15^N		
Age class	Sex	Mean	SD	Mean	SD	Mean	SD	Mean	SD	*n*
Infant/juvenile	All	46.15	3.00	3.22	1.00	−30.01	0.70	2.05	0.46	13
	Female	46.92	2.84	2.92	0.81	−30.22	0.62	2.01	0.36	10
	Male	43.84	2.56	4.19	1.05	−29.40	0.65	2.18	0.80	3
Adolescent	All	48.05	2.13	3.52	0.98	−30.29	0.76	1.88	0.89	15
	Female	48.04	1.83	3.37	1.00	−30.46	0.63	2.41	0.72	7
	Male	48.05	2.48	3.65	1.01	−30.14	0.87	1.42	0.79	8
Adult	All	47.00	3.28	3.26	1.12	−30.15	0.75	2.15	0.93	38
	Male	47.78	3.43	3.28	1.08	−30.01	0.69	2.07	0.91	22
	Female	45.86	2.77	3.24	1.21	−30.33	0.82	2.26	0.96	16
Total	All	47.09	3.02	3.31	1.06	−30.16	0.74	2.07	0.84	66
	Female	47.63	3.03	3.20	0.99	−30.14	0.67	2.11	0.77	39
	Male	46.30	2.89	3.47	1.14	−30.17	0.84	2.00	0.95	27

**FIGURE 3:**
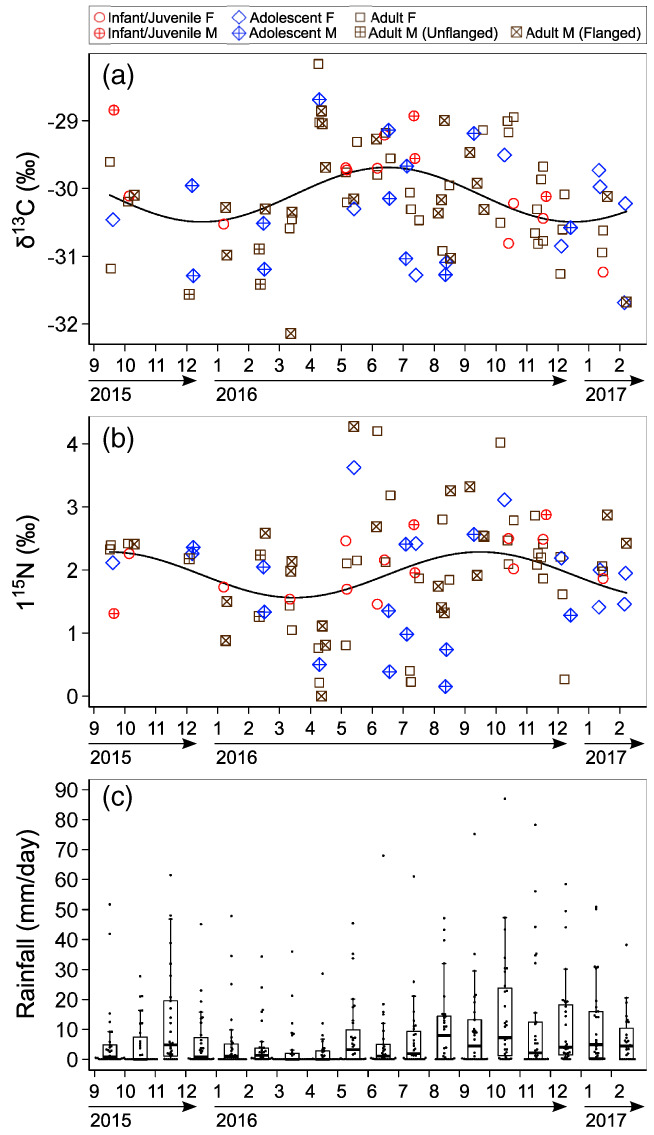
Fluctuations in non‐averaged (a) δ^13^C and (b) δ^15^N values of fecal samples from wild orangutans in Danum valley conservation area. The solid lines represent the estimated relationship between fecal stable isotope ratios and the date of the year based on the best linear mixed‐effects models. (c) Boxplot and raw data of the daily amount of rainfall are shown for each month^1^

No significant effect was seen in the age and sex as explanatory variables for the non‐averaged orangutan fecal stable isotope ratios, but the sine and cosine of the sampling date had a significant effect (Table 4; Figure 3). It seems that the lowest fecal δ^13^C values were seen after the months with the highest daily amount of rainfall, November 2015 and October 2016 (Figure 3).

**TABLE 4:** Explanatory variables and intercept in the linear mixed models for fecal elemental concentration and stable isotope ratios of wild orangutans in Danum valley conservation area

Response variables	Explanatory variables		Effect	SE	t	*p*‐value
%C	Fixed effect	**Intercept**	**47.49**	**1.38**	**34.35**	**<0.001**
		Sex: Male	−0.42	1.80	−0.23	0.816
		Age: Adult	0.31	1.56	0.20	0.843
		Age: Infant/juvenile	−0.54	1.90	−0.28	0.777
		Date: Sine	0.37	0.60	0.61	0.544
		Date: Cosine	0.61	0.64	0.95	0.344
		Sex: Male × Age: Adult	−2.45	2.13	−1.15	0.253
		Sex: Male × Age: Infant/juvenile	−3.70	3.02	−1.22	0.224
	Random effect	SD of ndividual ID	0.00	0.00	–	–
%N	Fixed effect	**Intercept**	**3.48**	**0.48**	**7.25**	**<0.001**
		Sex: Male	0.25	0.62	0.41	0.6899
		Age: Adult	−0.22	0.55	−0.40	0.6931
		Age: Infant/juvenile	−0.58	0.62	−0.94	0.3621
		Date: Sine	−0.23	0.16	−1.47	0.1445
		Date: Cosine	0.30	0.17	1.76	0.0829
		Sex: Male × Age: Adult	−0.17	0.74	−0.24	0.8172
		Sex: Male × Age: Infant/Juvenile	0.69	1.01	0.68	0.5062
	Random effect	SD of ndividual ID	0.23	0.48	–	–
δ^13^C	Fixed effect	**Intercept**	**−30.24**	**0.33**	**−92.96**	**<0.001**
		Sex: Male	0.05	0.42	0.12	0.908
		Age: Adult	0.37	0.37	0.98	0.346
		Age: Infant/juvenile	0.11	0.42	0.26	0.800
		**Date: Sine**	**−0.40**	**0.11**	**−3.71**	**<0.001**
		Date: Cosine	0.03	0.12	0.23	0.822
		Sex: Male × Age: Adult	−0.47	0.50	−0.93	0.367
		Sex: Male × Age: Infant/juvenile	0.63	0.69	0.92	0.374
	Random effect	SD of individual ID	0.10	0.31	–	–
δ^15^N	Fixed effect	Intercept	2.25	0.30	7.51	0.088
		Sex: Male	−0.86	0.39	−2.20	0.267
		Age: Adult	−0.35	0.34	−1.04	0.511
		Age: Infant/juvenile	−0.23	0.40	−0.59	0.641
		Date: Sine	0.06	0.12	0.52	0.603
		**Date: Cosine**	**0.37**	**0.13**	**2.86**	**0.009**
		Sex: Male × Age: Adult	1.04	0.46	2.26	0.257
		Sex: Male × Age: Infant/juvenile	0.86	0.64	1.33	0.360
	Random effect	SD of individual ID	0.02	0.15	–	–

*Note*: Significant fixed effects are shown in bold.

**FIGURE 4:**
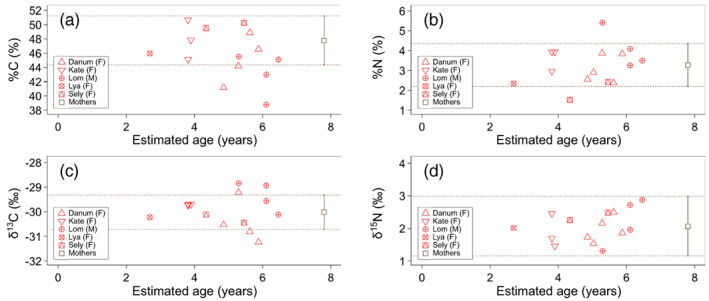
Age change in non‐averaged (a) %C, (b) %N, (c) δ^13^C, and (d) δ^15^N values of infant/juvenile fecal samples. Raw data were used instead of average data of infant/juvenile fecal samples collected from the same individual during the same month. Means ± *SD* of maternal values are shown with dotted lines

Mann–Whitney U tests indicated no significant difference in fecal %C (U = 100, *p* = 0.261), %N (U = 137, *p* = 0.853), δ^13^C (U = 134, *p* = 0.958), and δ^15^N (U = 136, *p* = 0.827) values between the infant/juveniles (2.7–6.5 years) and mothers (Figure 3; Figure S3). Fecal %C, %N, δ^13^C, and δ^15^N values in infant/juveniles were mostly within one SD of maternal values (Figure 4).

Neither age class, sex, nor their interactions had a significant effect as an explanatory variable for the non‐averaged orangutan fecal elemental concentrations (Table 4).

### 3.3 Comparison with fossil δ^13^C values

**FIGURE 5:**
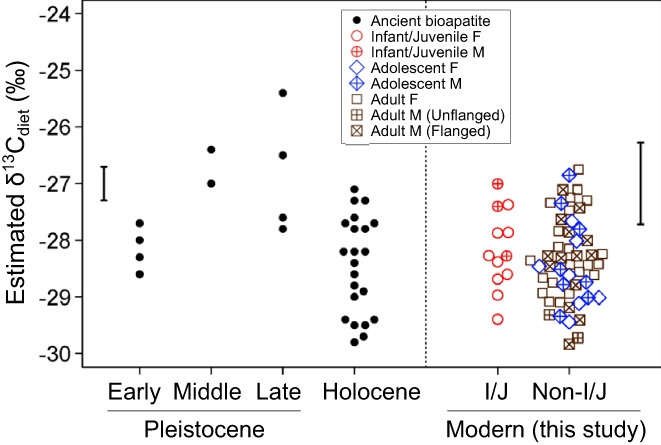
Bee swarm plot showing estimated dietary δ^13^C values calculated from the δ^13^C values of bioapatite for fossil orangutans and δ^13^C values of feces for infant/juvenile (I/J) and the other (non‐I/J) modern orangutans in Danum valley conservation area. The Suess effect was considered, and 1.5‰ was subtracted from δ^13^C values of fossil specimens (Friedli et al., 1986) to compare modern fecal δ^13^C with fossil δ^13^C values. Typical error ranges of isotopic offsets between diet and apatite (Malone et al., 2021) and between diet and feces (Tsutaya, Ogawa, et al., 2021) are also shown

Dietary δ^13^C values of extant and fossil orangutans are significantly elevated during the Middle to Late Pleistocene (Figure 5). A Kruskal–Wallis test indicated a significant difference in dietary δ^13^C values calculated from fossil bioapatite and modern infant/juvenile fecal samples (χ^2^ = 12.0, *p* = 0.017). Fecal δ^13^C values obtained from infant/juvenile individuals in DVCA were used for comparison because enamel δ^13^C values represent dietary input during orangutan tooth formation from ~0 to 8 years of age (Beynon et al., 1991). Post hoc Mann–Whitney *U*‐tests with a corrected *p*‐value of 0.0083 (= 0.05/6), using the Bonferroni method (Bland & Altman, 1995), indicated significant differences between dietary δ^13^C values from Middle to Late Pleistocene and Holocene (difference = 1.8‰, U = 127.5, *p* = 0.0018) and Middle to Late Pleistocene and Modern (difference = 1.4‰, U = 66.0, *p* = 0.0032). No significant difference was seen between dietary δ^13^C values calculated from Holocene and Modern samples (U = 112.0, *p* = 0.3752).

## 4 DISCUSSION

### 4.1 Plant foods

Plant parts had no significant effect on the variation of plant δ^13^C and δ^15^N values (Table 2; Figures 1 and 2). This result in the DVCA does not support Hypothesis 1 (isotopic variation among plant parts) and contradicts empirical results from stable isotopic research on plants eaten by African great apes that showed significant δ^13^C differences between fruits and leaves (e.g., Blumenthal et al., 2012; Oelze et al., 2014). Investigation of the cause of this result is beyond the scope of this study, and systematic sample collection of plant parts at different tree heights and during different periods is needed to elucidate this unexpected result further.

Orangutans are arboreal animals, rarely moving to the ground, and thus consume plant foods higher in the canopy (Kanamori et al., 2010, but see Ancrenaz et al., 2014). All plant food samples were collected from the same tree height as orangutan feeding height in this study, and only plants from higher in the canopy were sampled, except for ginger as discussed below. Thus, plant food δ^13^C did not show the lower values that are typical of the understory in closed‐canopy forests (Medina et al., 1991; van der Merwe & Medina, 1991). The only exception to this explanation is ginger (*Etlingera megalocheilos*), an understory plant sometimes consumed by orangutans. A single data point for ginger is included in this data set, but its δ^13^C value (−28.9‰) is rather greater than the overall mean δ^13^C value of plant samples (−30.3 ± 1.4 ‰). A canopy effect is lacking for ginger or perhaps absent in the DVCA. Other understory plants were not sampled, and these alternatives cannot be distinguished.

### 4.2 Orangutan feces

The LMMs did not support Hypothesis 2 (individual variation in fecal isotope ratios), and homogenous stable isotope ratios of food sources observed in the DVCA limits the utility of stable isotope analysis to differentiate intrapopulation dietary differences in a primary forest. We did not find systematic variations in δ^13^C and δ^15^N values of feces by sex and age class of orangutan individuals (Table 4; Figures 1 and 2), perhaps reflecting homogenous δ^13^C and δ^15^N values in plants. Even if dietary preferences significantly differ among individual orangutans, these differences cannot be detected through stable isotope analysis when isotope ratios vary little among food sources.

In contrast, the LMMs supported Hypothesis 3 (seasonal variation in fecal isotope ratios), and the periodic fluctuations in δ^13^C and δ^15^N values of orangutan feces were found (Table 4; Figure 3). Such an intra‐annual fluctuation could be the result of seasonal fluctuations in plant food baseline isotope data. However, we did not observe any effect of the rainfall period on our plant food isotope data set. Another explanation for seasonal fluctuations in fecal isotope ratios could be fluctuations in plant foods consumed. However, observational data revealed that orangutans in DVCA intensively fed on a relatively limited number of plant taxa both during lower and higher fruiting months (Kanamori et al., 2010). Therefore, the possibility of a large contribution from unanalyzed seasonal food sources is unlikely. Since the plant δ^13^C values decrease in wet and humid environments (Heaton, 1999), the observed decrease in fecal δ^13^C values just after the months with the highest amount of daily rainfall is consistent with the scenario of fluctuation in baseline isotope ratios (Figure 3). However, we were not able to investigate this issue further due to the lack of year‐round data of plant isotope ratios. It is necessary to analyze δ^13^C and δ^15^N values of food plants continuously over a few years to evaluate the impact of changes in rainfall on baseline plant stable isotope ratios. In addition, the relationship between stable isotope ratios of plants and phenology needs to be investigated in a future study.

Fecal %C, %N, δ^13^C, and δ^15^N values do not differ significantly between the one infant, as well as juveniles older than 2.7 years old, and their mothers (Figure 4). Therefore, Hypothesis 4 (isotopic effect of breastfeeding) was not supported in this study, though several previous studies showed a systematic increase in %N, δ^13^C, and δ^15^N values in breastfed infants of chimpanzee and langur (Bădescu et al., 2017, 2022; Reitsema, 2012; but see Reitsema et al., 2020). Behavioral and trace elemental studies showed that breastfeeding continues ~7 years after birth in wild orangutans (Mendonça et al., 2017; Smith et al., 2017; van Noordwijk et al., 2009, 2013; van Noordwijk & van Schaik, 2005), and orangutan infant/juveniles investigated in this study, aged from 2.7 to 6.5 years, are expected to consume breast milk. The unexpected finding of no significant differences in %C, %N, δ^13^C, and δ^15^N between infant/juvenile and mother feces might result from the inclusion of many samples from juveniles and/or a discrepancy between suckling frequency and breast milk consumption. Although younger individuals are expected to record a stronger signal of breast milk intake, the youngest infant/juveniles' age at the fecal sample collection was 2.7 years, and the second youngest age was 3.8 years in our data set. It is highly likely that breastfeeding signals can be seen in the fecal samples from infants younger than 2.7 years because of the relatively higher frequency of suckling and larger nutrient contribution of breast milk in their diet (Mendonça et al., 2017; van Noordwijk et al., 2013), although such samples are not included in this study. In addition, the isotopic contribution of breast milk may be fairly small in juvenile orangutans older than 3–4 years, while they suckle the mother's nipple until the age of 7 years. However, identifying a breastfeeding and weaning pattern using fecal stable isotope ratios typically requires hundreds of matched infant and maternal fecal specimens across the entire infancy to uncover any true breast milk signal masked by a large amount of noise (Bădescu et al., 2017, 2022; Reitsema, 2012), and only a few samples were matched specimens in our data set (Figure S3). Orangutans are semi‐solitary, and the collection of a large number of small infant fecal samples from wild orangutans is cost‐intensive compared with collection from group‐living African great apes. Fecal proteomics would be a more feasible method to investigate breast milk consumption from a smaller number of samples (Tsutaya, Mackie, et al., 2021).

Carbon and nitrogen elemental concentrations of feces did not systematically vary with sex and age class in orangutans in the DVCA (Table 3; Figures 1 and 2), and thus Hypothesis 5 (variation in elemental concentrations of feces) was not supported. This result suggests that the combined effect of the elemental difference of different plant parts and individual dietary differences were not large enough to be detected in the elemental concentration of feces. Consumption of several different food categories with different proportions will affect the elemental concentration of feces if the elemental concentrations of these food categories are sufficiently variable (Reitsema et al., 2020). Flanged male orangutans spend a greater amount of feeding time on leaves (60.3%) compared with unflanged males, adult females, and adolescent females in DVCA (22.4%–24.9%: Kanamori et al., 2010). Dietary leaves show greater %N than fruits and bark (Table 1; Figure 1), and thus fecal %N values of flanged male orangutans are expected to be greater than those of other individuals. In fact, however, this expectation was not the case (Supplementary Text 1.4 in Data S1). Feces consist of food remains, intestinal bacteria, and exfoliated host cells. Since the elemental concentrations of intestinal bacteria and host cells are relatively stable compared with food items, changes in elemental concentrations associated with diet would be largely masked in feces.

### 4.3 Relevance in palaeoenvironmental reconstruction

Comparison of fecal δ^13^C values of extant orangutans and apatite δ^13^C values of fossil orangutans revealed a significant increase in dietary δ^13^C values of orangutans in Middle and Late Pleistocene and similar dietary δ^13^C values of orangutans in Early Pleistocene, Holocene, and the modern DVCA (Figure 5). Orangutans were likely living in habitats similar to the modern‐day DVCA, a primary lowland dipterocarp forest, during the Early Pleistocene and Holocene, but were living in a relatively more arid or open habitat during the Middle and Late Pleistocene, where plants typically show greater δ^13^C values. Several records of paleoenvironmental proxies suggest that precipitation lessened and drier grassland environments expanded throughout Southeast Asia during the Middle and Late Pleistocene, due to a lower sea level and resultant severe environmental change in the region (Bird et al., 2005; Louys & Roberts, 2020; Wurster et al., 2019). Habitats of orangutans contracted, and orangutans were forced to subsist in more open landscapes compared with present primary rainforests (Louys & Roberts, 2020). This study supports this hypothesis since dietary δ^13^C values of fossil orangutans during the Middle and Late Pleistocene were ~1.4‰ higher than those found in extant orangutans (Figure 5). Stable isotope ratios of wild orangutans from a known ecological context helps in the interpretation of δ^13^C values obtained from fossil orangutans.

However, the comparative framework employed here is limited due to differences in tissues used for isotope analysis. Tissue δ^13^C values were converted to dietary δ^13^C values by applying tissue‐specific isotopic offset values, adding uncertainty for comparison of fecal and apatite δ^13^C values. Stable isotopic offset values from diet to body tissues have an error range of a few per thousand and vary due to several factors, such as macronutrient composition of the diet, the composition of intestinal microbiota, nutritional status, and digestive physiology (Crowley et al., 2010; Malone et al., 2021). Ideally, the same tissue (i.e., bioapatite) would be used for stable isotope comparisons between extant and fossil orangutans, as has been done for chimpanzees (Nelson, 2013).

### 4.4 Implications for conservation of orangutans

Carbon and nitrogen isotope ratios of foods and feces of wild orangutans in DVCA were not useful for examining intrapopulation differences in their diet but may be useful for assessing anthropogenic effects on the diet. δ^13^C values of orangutan foods in the primary rainforest were consistent with expectations for canopy C_3_ plants in a closed‐canopy rainforest (Hyodo et al., 2010). In contrast, δ^13^C values from understory plants were typically less than in canopy plants in the same forest due to the canopy effect (Medina et al., 1991; van der Merwea & Medinab, 1991). Moreover, plant δ^13^C values from open habitats and C_4_ ecosystems are typically greater than those from closed forests and C_3_ ecosystems (Heaton, 1999; van der Merwe & Medina, 1989; van der Merwea & Medinab, 1991). Thus, an increase in terrestrial movements inside a closed‐canopy forest and consumption of foods from the understory (Ancrenaz et al., 2014) is anticipated to increase δ^13^C values. In addition, foods from more open landscapes (Ancrenaz et al., 2015), such as plantations and logged forests, or C_4_ agricultural products, should be easily detected as elevated δ^13^C values in orangutan tissues using in these habitats. δ^15^N values in plants increase with the application of organic fertilizers, such as animal manure and food wastes (Szpak, 2014), and consumption of fertilized agricultural products can also be detected from elevated δ^15^N values.

Anthropogenic degradation of forest habitats, as well as hunting, is a substantial threat to extant orangutans (e.g., Freund et al., 2017; Gaveau et al., 2014; Meijaard et al., 2011; Sabah Wildlife Department, 2020; Wich et al., 2012), and characterization of orangutan feeding ecology in degraded landscapes is essential for more efficient conservation of the species. Growing numbers of conflicts between orangutans and humans, such as raiding of agricultural products by orangutans and killing of orangutans by humans, are a consequence of the expansion of agricultural landscapes, plantations, and logged areas (Freund et al., 2017; Meijaard et al., 2011). These anthropogenic impacts on orangutan habitats and diet can be monitored more efficiently and easily through stable isotope analysis. Such an application is possible given the baseline stable isotope ratios presented in this study for orangutans and their foods in a minimally disturbed primary rainforest.

### 4.5 Limitations of this study and future directions

There are several limitations in this study that await further investigation in future studies. First, the present study is based on a single wild orangutan population in a lowland primary dipterocarp forest with mast fruiting. However, feeding ecology inferred from stable isotopes might differ in other types of forest, such as peat swamp, highland, or non‐mast fruiting forests. Second, data were not obtained during a mast fruiting period in this study. Understanding stable isotope ecology during mast fruiting periods will produce a more comprehensive understanding of orangutan feeding ecology. Third, there are other variables that are worth being investigated in relation to the fecal isotope ratios. For example, detailed analysis of behavioral data would enable a more direct comparison of the stable isotope ratios and elemental concentrations of feces and those of what the orangutan individuals were eating at the time of sample collection. In addition, temporal fluctuations in the stable isotope ratios of feces should be compared with the phenology because seasonal fluctuations in orangutans' dietary composition correlate with the fruit availability (Kanamori et al., 2010). Finally, although the potential utility of the stable isotope analysis to estimate the anthropogenic impact on orangutans' feeding behaviors was suggested in this study, the typical δ^13^C and δ^15^N values of food sources originating from degraded open habitats, farmlands, and plantations are unknown. It is necessary to confirm the δ^13^C and δ^15^N values of anthropogenic food sources are significantly different from those of food sources in primary forests.

## 5 CONCLUSIONS

Carbon and nitrogen stable isotope analysis was applied to feces and plant foods of wild Bornean orangutans in DVCA to characterize their feeding ecology. Stable carbon and nitrogen isotope ratios of plant species consumed by wild Bornean orangutans were homogenous, contrary to Hypothesis 1 (isotopic variation among plant parts). No significant differences were observed in %C, %N, δ^13^C, and δ^15^N values of orangutan fecal samples among different sex and age classes, nor between infant/juveniles (aged from 2.7 to 6.5 years) and mothers, which is contrary to Hypotheses 2 (individual variation in fecal isotope ratios), 4 (isotopic effect of breastfeeding), and 5 (variation in elemental concentrations of feces). However, fecal δ^13^C and δ^15^N values fluctuated with rainfall, which is consistent with Hypothesis 3 (seasonal variation in fecal isotope ratios). Several other aspects of isotopic ecology in orangutans, such as data from other types of habitats, data from mast fruiting periods, relationship with behavioral and ecological factors, a large sample size of orangutan age‐sex classes, and anthropogenic impacts, need to be investigated further to evaluate the usefulness of stable isotope analysis in ecological, evolutionary, and conservation studies of wild orangutans.

## AUTHOR CONTRIBUTIONS

**Takumi Tsutaya:** Conceptualization (lead); formal analysis (lead); funding acquisition (equal); investigation (equal); methodology (equal); project administration (lead); visualization (lead); writing – original draft (lead); writing – review and editing (lead). **Anna Wong:** Conceptualization (supporting); project administration (supporting); resources (equal); writing – original draft (supporting). **Peter T. Malim:** Conceptualization (equal); resources (equal). **Henry Bernard:** Conceptualization (equal); resources (equal); writing – original draft (supporting). **Nanako O. Ogawa:** Funding acquisition (equal); investigation (equal); visualization (supporting); writing – original draft (supporting). **Naohiko Ohkouchi:** Funding acquisition (equal); investigation (equal); validation (supporting); writing – original draft (equal). **Shun Hongo:** Formal analysis (equal); visualization (supporting); writing – review and editing (equal). **Tomoyuki Tajima:** Funding acquisition (equal); resources (equal); writing – original draft (supporting); writing – review and editing (supporting). **Tomoko Kanamori:** Conceptualization (equal); data curation (equal); funding acquisition (equal); methodology (equal); resources (equal); writing – original draft (supporting); writing – review and editing (supporting). **Noko Kuze:** Conceptualization (equal); data curation (equal); funding acquisition (equal); methodology (equal); resources (equal); writing – original draft (supporting); writing – review and editing (equal).

## CONFLICT OF INTEREST

The authors declare no conflicts of interest.

## Supporting information


**Supplementary Figure S1** Comparison of (a) %C, (b) %N, (c) δ^13^C, and (d) δ^15^N values of paired plant samples obtained from the same species + parts and collected during different periods.
**Supplementary Figure S2**. Comparison of plant (a) %C, (b) %N, (c) δ^13^ C, and (d) δ^15^N values obtained from different parts of the same species.
**Supplementary Figure S3**. Age change in infant/juvenile–mother difference in (a) %C, (b) %N, (c) δ^13^ C, and (d) δ^15^N values of the paired infant/juvenile–mother fecal samples collected within the same month. Raw data were used instead of average data of infant fecal samples collected from the same individual during the same month.
**Supplementary Table S1**. Individual information of the subject orangutans in this study. The estimated age of orangutan individuals, as of September 1st, 2015, with information on the relatively precise birth date.
**Supplementary Table S4**. Summary of the plant samples collected during different periods.
**Supplementary Table S5**. Results of the paired Mann–Whitney U tests for the same plant species + parts collected in different periods.
**Supplementary Table S6**. Explanatory variables and intercept in the linear mixed models for fecal %N of wild orangutans in DVCA. Significant fixed effects are shown in bold.

**Supplementary Table S2** Carbon and nitrogen stable isotope ratios of the analyzed fecal samples. Excluded samples as an outlier are marked with an asterisk.

**Supplementary Table S3** Carbon and nitrogen stable isotope ratios of the analyzed food samples. Orangutan individual that consumed the plant sample was also shown. Excluded samples as an outlier are marked with an asterisk.

## Data Availability

The data that support the findings of this study are openly available in the supplementary materials of this article.

## Endnote

^1^
Available via the following URL. Accessed on November 12, 2020. https://www.searrp.org/scientists/available‐data/.

## References

[ajpa24598-bib-0001] Ancrenaz, M. , Ambu, L. , Sunjoto, I. , Ahmad, E. , Manokaran, K. , Meijaard, E. , & Lackman, I. (2010). Recent surveys in the forests of Ulu Segama Malua, Sabah, Malaysia, show that orang‐utans (*P. p. morio*) can be maintained in slightly logged forests. PLoS One, 5, e11510. 20634974 10.1371/journal.pone.0011510 PMC2901384

[ajpa24598-bib-0002] Ancrenaz, M. , Gumal, M. , Marshall, A. J. , Meijaard, E. , Wich, S. A. , & Husson, S. (2016). Pongo pygmaeus, Bornean orangutan (Vol. 8235, pp. 2011–2016). International Union for Conservation of Nature.

[ajpa24598-bib-0003] Ancrenaz, M. , Oram, F. , Ambu, L. , Lackman, I. , Ahmad, E. , Elahan, H. , Kler, H. , Abram, N. K. , & Meijaard, E. (2015). Of *Pongo*, palms and perceptions: A multidisciplinary assessment of Bornean orang‐utans *Pongo pygmaeus* in an oil palm context. Oryx, 49, 465–472.

[ajpa24598-bib-0004] Ancrenaz, M. , Sollmann, R. , Meijaard, E. , Hearn, A. J. , Ross, J. , Samejima, H. , Loken, B. , Cheyne, S. M. , Stark, D. J. , Gardner, P. C. , Goossens, B. , Mohamed, A. , Bohm, T. , Matsuda, I. , Nakabayasi, M. , Lee, S. K. , Bernard, H. , Brodie, J. , Wich, S. , … Wilting, A. (2014). Coming down from the trees: Is terrestrial activity in Bornean orangutans natural or disturbance driven? Scientific Reports, 4, 4024. 24526001 10.1038/srep04024 PMC3923384

[ajpa24598-bib-0005] Bacon, A. M. , Bourgon, N. , Dufour, E. , Zanolli, C. , Duringer, P. , Ponche, J. L. , Antoine, P. O. , Shackelford, L. , Huong, N. T. M. , Sayavonkhamdy, T. , Patole‐Edoumba, E. , & Demeter, F. (2018). Nam lot (MIS 5) and Duoi U'Oi (MIS 4) southeast Asian sites revisited: Zooarchaeological and isotopic evidences. Palaeogeography Palaeoclimatology Palaeoecology, 512, 132–144.

[ajpa24598-bib-0006] Bădescu, I. , Katzenberg, M. A. , Watts, D. P. , & Sellen, D. W. (2017). A novel fecal stable isotope approach to determine the timing of age‐related feeding transitions in wild infant chimpanzees. American Journal of Physical Anthropology, 162, 285–299. 27768227 10.1002/ajpa.23116

[ajpa24598-bib-0007] Bădescu, I. , Watts, D. P. , Katzenberg, M. A. , & Sellen, D. W. (2022). Maternal lactational investment is higher for sons in chimpanzees. Behavioral Ecology and Sociobiology, 76, 44.

[ajpa24598-bib-0008] Bates, D. , Mächler, M. , Bolker, B. M. , & Walker, S. C. (2015). Fitting linear mixed‐effects models using lme4. Journal of Statistical Software, 67, 1–48.

[ajpa24598-bib-0009] Benner, R. , Fogel, M. L. , Sprague, E. K. , & Hodson, R. E. (1987). Depletion of C in lignin and its implications for stable carbon isotope studies. Nature, 329, 708–710.

[ajpa24598-bib-0010] Beynon, A. D. , Dean, M. C. , & Reid, D. J. (1991). Histological study on the chronology of the developing dentition in gorilla and orangutan. American Journal of Physical Anthropology, 86, 189–203.

[ajpa24598-bib-0011] Bird, M. I. , Taylor, D. , & Hunt, C. (2005). Palaeoenvironments of insular Southeast Asia during the last glacial period: A savanna corridor in Sundaland? Quaternary Science Reviews, 24, 2228–2242.

[ajpa24598-bib-0012] Bland, J. M. , & Altman, D. G. (1995). Multiple significance tests: The Bonferroni method. British Medical Journal, 310, 170. 7833759 10.1136/bmj.310.6973.170 PMC2548561

[ajpa24598-bib-0013] Blumenthal, S. A. , Chritz, K. L. , Rothman, J. M. , & Cerling, T. E. (2012). Detecting intraannual dietary variability in wild mountain gorillas by stable isotope analysis of feces. Proceedings of the National Academy of Sciences, 109, 21277–21282. 10.1073/pnas.1215782109 PMC3535629 23236160

[ajpa24598-bib-0014] Blumenthal, S. A. , Rothman, J. M. , Chritz, K. L. , & Cerling, T. E. (2016). Stable isotopic variation in tropical forest plants for applications in primatology. American Journal of Primatology, 78, 1041–1054. 26444915 10.1002/ajp.22488

[ajpa24598-bib-0015] Bocherens, H. , Schrenk, F. , Chaimanee, Y. , Kullmer, O. , Mörike, D. , Pushkina, D. , & Jaeger, J. J. (2017). Flexibility of diet and habitat in Pleistocene south Asian mammals: Implications for the fate of the giant fossil ape Gigantopithecus. Quaternary International, 434, 148–155.

[ajpa24598-bib-0016] Bogaard, A. , Fraser, R. , Heaton, T. H. E. , Wallace, M. , Vaiglova, P. , Charles, M. , Jones, G. , Evershed, R. P. , Styring, A. K. , Andersen, N. H. , Arbogast, R. M. , Bartosiewicz, L. , Gardeisen, A. , Kanstrup, M. , Maier, U. , Marinova, E. , Ninov, L. , Schäfer, M. , & Stephan, E. (2013). Crop manuring and intensive land management by Europe's first farmers. Proceedings of the National Academy of Sciences, 110, 12589–12594. 10.1073/pnas.1305918110 PMC3732975 23858458

[ajpa24598-bib-0017] Campbell‐Smith, G. , Campbell‐Smith, M. , Singleton, I. , & Linkie, M. (2011a). Raiders of the lost bark: Orangutan foraging strategies in a degraded landscape. PLoS One, 6, e20962. 21731636 10.1371/journal.pone.0020962 PMC3120831

[ajpa24598-bib-0018] Campbell‐Smith, G. , Campbell‐Smith, M. , Singleton, I. , & Linkie, M. (2011b). Apes in space: Saving an imperilled orangutan population in Sumatra. PLoS One, 6, e17210. 21364732 10.1371/journal.pone.0017210 PMC3040220

[ajpa24598-bib-0019] Cernusak, L. , Tcherkez, G. , & Keitel, C. (2009). Why are non‐photosynthetic tissues generally ^13^C enriched compared with leaves in C_3_ plants? Review and synthesis of current hypotheses. Functional Plant Biology, 36, 199–213. 32688639 10.1071/FP08216

[ajpa24598-bib-0020] Crowley, B. E. (2012). Stable isotope techniques and applications for primatologists. International Journal of Primatology, 33, 673–701.

[ajpa24598-bib-0021] Crowley, B. E. , Carter, M. L. , Karpanty, S. M. , Zihlman, A. L. , Koch, P. L. , & Dominy, N. J. (2010). Stable carbon and nitrogen isotope enrichment in primate tissues. Oecologia, 164, 611–626. 20628886 10.1007/s00442-010-1701-6 PMC2955919

[ajpa24598-bib-0022] Dawson, T. E. , Mambelli, S. , Plamboeck, A. H. , Templer, P. H. , & Tu, K. P. (2002). Stable isotopes in plant ecology. Annual Review of Ecology and Systematics, 33, 507–559.

[ajpa24598-bib-0023] Eckrich, C. A. , Albeke, S. E. , Flaherty, E. A. , & Ben‐david, R. T. B. M. (2020). rKIN: Kernel‐based method for estimating isotopic niche size and overlap. The Journal of Animal Ecology, 89, 757–771. 31799690 10.1111/1365-2656.13159

[ajpa24598-bib-0024] Evans, R. D. (2001). Physiological mechanisms influencing plant nitrogen isotope composition. Trends in Plant Science, 6, 121–126. 11239611 10.1016/s1360-1385(01)01889-1

[ajpa24598-bib-0025] Fahy, G. E. , Richards, M. , Riedel, J. , Hublin, J.‐J. , & Boesch, C. (2013). Stable isotope evidence of meat eating and hunting specialization in adult male chimpanzees. Proceedings of the National Academy of Sciences, 110, 5829–5833. 10.1073/pnas.1221991110 PMC3625252 23530185

[ajpa24598-bib-0026] Fahy, G. E. , Richards, M. P. , Fuller, B. T. , Deschner, T. , Hublin, J.‐J. , & Boesch, C. (2014). Stable nitrogen isotope analysis of dentine serial sections elucidate sex differences in weaning patterns of wild chimpanzees (*pan troglodytes*). American Journal of Physical Anthropology, 153, 635–642. 24395019 10.1002/ajpa.22464

[ajpa24598-bib-0027] Freund, C. , Rahman, E. , & Knott, C. (2017). Ten years of orangutan‐related wildlife crime investigation in West Kalimantan, Indonesia. American Journal of Primatology, 79, 22620. 10.1002/ajp.22620 27960033

[ajpa24598-bib-0028] Friedli, H. , Lötscher, H. , Oeschger, H. , Siegenthaler, U. , & Stauffer, B. (1986). Ice core record of the ^13^C/^12^C ratio of atmospheric CO_2_ in the past two centuries. Nature, 324, 237–238.

[ajpa24598-bib-0029] Fuller, B. T. , Fuller, J. L. , Harris, D. A. , & Hedges, R. E. M. (2006). Detection of breastfeeding and weaning in modern human infants with carbon and nitrogen stable isotope ratios. American Journal of Physical Anthropology, 129, 279–293. 16261548 10.1002/ajpa.20249

[ajpa24598-bib-0030] Gaveau, D. L. A. , Sloan, S. , Molidena, E. , Yaen, H. , Sheil, D. , Abram, N. K. , Ancrenaz, M. , Nasi, R. , Quinones, M. , Wielaard, N. , & Meijaard, E. (2014). Four decades of forest persistence, clearance and logging on Borneo. PLoS One, 9, e101654. 25029192 10.1371/journal.pone.0101654 PMC4100734

[ajpa24598-bib-0031] Hanya, G. , Stevenson, P. , van Noordwijk, M. , Te Wong, S. , Kanamori, T. , Kuze, N. , Aiba, S. , Chapman, C. A. , & van Schaik, C. (2011). Seasonality in fruit availability affects frugivorous primate biomass and species richness. Ecography, 34, 1009–1017.

[ajpa24598-bib-0032] Heaton, T. H. E. (1999). Spatial, species, and temporal variations in the ^13^C/^12^C ratios of C_3_ plants: Implications for palaeodiet studies. Journal of Archaeological Science, 26, 637–649.

[ajpa24598-bib-0033] Hyodo, F. , Matsumoto, T. , Takematsu, Y. , Kamoi, T. , Fukuda, D. , Nakagawa, M. , & Itioka, T. (2010). The structure of a food web in a tropical rain forest in Malaysia based on carbon and nitrogen stable isotope ratios. Journal of Tropical Ecology, 26, 205–214.

[ajpa24598-bib-0034] Ibrahim, Y. K. , Tshen, L. T. , Westaway, K. E. , Cranbrook, E. , Humphrey, L. , Muhammad, R. F. , Zhao, J. x. , & Peng, L. C. (2013). First discovery of Pleistocene orangutan (*Pongo sp*.) fossils in peninsular Malaysia: Biogeographic and paleoenvironmental implications. Journal of Human Evolution, 65, 770–797. 24210657 10.1016/j.jhevol.2013.09.005

[ajpa24598-bib-0035] Kanamori, T. , Kuze, N. , Bernard, H. , Malim, T. P. , & Kohshima, S. (2010). Feeding ecology of Bornean orangutans (*Pongo pygmaeus morio*) in Danum Valley, Sabah, Malaysia: A 3‐year record including two mast fruitings. American Journal of Primatology, 72, 820–840. 20653008 10.1002/ajp.20848

[ajpa24598-bib-0036] Kanamori, T. , Kuze, N. , Bernard, H. , Malim, T. P. , & Kohshima, S. (2017). Fluctuations of population density in Bornean orangutans (*Pongo pygmaeus morio*) related to fruit availability in the Danum Valley, Sabah, Malaysia: A 10‐year record including two mast fruitings and three other peak fruitings. Primates, 58, 225–235. 27848156 10.1007/s10329-016-0584-5

[ajpa24598-bib-0037] Knott, C. D. (1998). Changes in orangutan caloric intake, energy balance, and ketones in response to fluctuating fruit availability. International Journal of Primatology, 19, 1061–1079.

[ajpa24598-bib-0038] Kohn, M. J. , & Cerling, T. E. (2002). Stable isotope compositions of biological apatite. Reviews in Mineralogy and Geochemistry, 48, 455–488.

[ajpa24598-bib-0039] Kuze, N. , Kawabata, H. , Yamazaki, S. , Kanamori, T. , Malim, T. P. , & Bernard, H. (2011). A wild Borneo orangutan carries large numbers of branches on the neck for feeding and nest building in the Danum Valley conservation area. Primate Research., 27, 21–26.

[ajpa24598-bib-0040] Kuze, N. , Malim, T. P. , & Kohshima, S. (2005). Developmental changes in the facial morphology of the Borneo orangutan (*Pongo pygmaeus*): Possible signals in visual communication. American Journal of Primatology, 65, 353–376. 15834889 10.1002/ajp.20121

[ajpa24598-bib-0041] Loader, N. J. , Robertson, I. , & McCarroll, D. (2003). Comparison of stable carbon isotope ratios in the whole wood, cellulose and lignin of oak tree‐rings. Palaeogeography Palaeoclimatology Palaeoecology, 196, 395–407.

[ajpa24598-bib-0042] Locke, D. P. , Hillier, L. W. , Warren, W. C. , Worley, K. C. , Nazareth, L. V. , Muzny, D. M. , Yang, S. P. , Wang, Z. , Chinwalla, A. T. , Minx, P. , Mitreva, M. , Cook, L. , Delehaunty, K. D. , Fronick, C. , Schmidt, H. , Fulton, L. A. , Fulton, R. S. , Nelson, J. O. , Magrini, V. , … Wilson, R. K. (2011). Comparative and demographic analysis of orang‐utan genomes. Nature, 469, 529–533. 21270892 10.1038/nature09687 PMC3060778

[ajpa24598-bib-0043] Loudon, J. E. , Grobler, J. P. , Sponheimer, M. , Moyer, K. , Lorenz, J. G. , & Turner, T. R. (2014). Using the stable carbon and nitrogen isotope compositions of vervet monkeys (*Chlorocebus pygerythrus*) to examine questions in ethnoprimatology. PLoS One, 9, e100758. 25010211 10.1371/journal.pone.0100758 PMC4091945

[ajpa24598-bib-0044] Loudon, J. E. , Sandberg, P. A. , Wrangham, R. W. , Fahey, B. , & Sponheimer, M. (2016). The stable isotope ecology of *pan* in Uganda and beyond. American Journal of Primatology, 78, 1070–1085. 27188271 10.1002/ajp.22552

[ajpa24598-bib-0045] Loudon, J. E. , Wakefield, M. L. , Kimel, H. M. , Waller, M. T. , Hickmott, A. J. , White, F. J. , & Sponheimer, M. (2019). Stable isotope data from bonobo (*Pan paniscus*) faecal samples from the Lomako Forest reserve, Democratic Republic of the Congo. African Journal of Ecology, 57, 437–442.

[ajpa24598-bib-0046] Louys, J. , & Roberts, P. (2020). Environmental drivers of megafauna and hominin extinction in Southeast Asia. Nature, 586, 402–406. 33029012 10.1038/s41586-020-2810-y

[ajpa24598-bib-0047] Lowry, B. E. , Wittig, R. M. , Pittermann, J. , & Oelze, V. M. (2021). Stratigraphy of stable isotope ratios and leaf structure within an African rainforest canopy with implications for primate isotope ecology. Scientific Reports, 11, 14222. 34244559 10.1038/s41598-021-93589-8 PMC8270916

[ajpa24598-bib-0048] Macharia, A. N. , Cerling, T. E. , Jorgensen, M. J. , & Kaplan, J. R. (2014). The hair‐diet δ^13^C and δ^15^N fractionation in *Chlorocebus aethiops sabaeus* based on a control diet study. Annales Zoologici Fennici, 51, 66–72.

[ajpa24598-bib-0049] Malone, M. A. , MacLatchy, L. M. , Mitani, J. C. , Kityo, R. , & Kingston, J. D. (2021). A chimpanzee enamel‐diet δ^13^C enrichment factor and a refined enamel sampling strategy: Implications for dietary reconstructions. Journal of Human Evolution, 159, 103062. 34536662 10.1016/j.jhevol.2021.103062 PMC8478842

[ajpa24598-bib-0050] Medina, E. , Sternberg, L. , & Cuevas, E. (1991). Vertical stratification of δ^13^C values in closed natural and plantation forests in the Luquillo mountains, Puerto Rico. Oecologia, 87, 369–372. 28313264 10.1007/BF00634593

[ajpa24598-bib-0051] Meijaard, E. , Albar, G. , Nardiyono, R. Y. , Ancrenaz, M. , & Spehar, S. (2010). Unexpected ecological resilience in Bornean orangutans and implications for pulp and paper plantation management. PLoS One, 5, e12813. 20877646 10.1371/journal.pone.0012813 PMC2943906

[ajpa24598-bib-0052] Meijaard, E. , Buchori, D. , Hadiprakarsa, Y. , Utami‐Atmoko, S. S. , Nurcahyo, A. , Tjiu, A. , Prasetyo, D. , Nardiyono, C. L. , Ancrenaz, M. , Abadi, F. , Antoni, I. N. G. , Armayadi, D. , Dinato, A. , Ella, G. , Indrawan, P. , Kussaritano, T. P. , Munajat, C. , Priyono, C. W. P. , Purwanto, Y. , … Mengersen, K. (2011). Quantifying killing of orangutans and human‐orangutan conflict in Kalimantan, Indonesia. PLoS One, 6, e27491. 22096582 10.1371/journal.pone.0027491 PMC3214049

[ajpa24598-bib-0053] Meijaard, E. , Ni'matullah, S. , Dennis, R. , Sherman, J. , Onrizal , & Wich, S. A. (2021). The historical range and drivers of decline of the Tapanuli orangutan. PLoS One, 16, e0238087. 33395430 10.1371/journal.pone.0238087 PMC7781382

[ajpa24598-bib-0054] Meijaard, E. , Welsh, A. , Ancrenaz, M. , Wich, S. , Nijman, V. , & Marshall, A. J. (2010). Declining orangutan encounter rates from Wallace to the present suggest the species was once more abundant. PLoS One, 5, e12042. 20711451 10.1371/journal.pone.0012042 PMC2920314

[ajpa24598-bib-0055] Meijaard, E. , Wich, S. , Ancrenaz, M. , & Marshall, A. J. (2012). Not by science alone: Why orangutan conservationists must think outside the box. Annals of the New York Academy of Sciences, 1249, 29–44. 22175247 10.1111/j.1749-6632.2011.06288.x

[ajpa24598-bib-0056] Mendonça, R. S. , Kanamori, T. , Kuze, N. , Hayashi, M. , Bernard, H. , & Matsuzawa, T. (2017). Development and behavior of wild infant‐juvenile east Bornean orangutans (*Pongo pygmaeus morio*) in Danum Valley. Primates, 58, 211–224. 27600514 10.1007/s10329-016-0567-6

[ajpa24598-bib-0057] Mendonça, R. S. , Takeshita, R. S. C. , Kanamori, T. , Kuze, N. , Hayashi, M. , Kinoshita, K. , Bernard, H. , & Matsuzawa, T. (2016). Behavioral and physiological changes in a juvenile Bornean orangutan after a wildlife rescue. Global Ecology and Conservation, 8, 116–122.

[ajpa24598-bib-0058] Minagawa, M. , & Wada, E. (1984). Stepwise enrichment of δ^15^N along food chains: Further evidence and the relation between δ^15^N and animal age. Geochimica et Cosmochimica Acta, 48, 1135–1140.

[ajpa24598-bib-0059] Nater, A. , Mattle‐Greminger, M. P. , Nurcahyo, A. , Nowak, M. G. , de Manuel, M. , Desai, T. , Groves, C. , Pybus, M. , Sonay, T. B. , Roos, C. , Lameira, A. R. , Wich, S. A. , Askew, J. , Davila‐Ross, M. , Fredriksson, G. , de Valles, G. , Casals, F. , Prado‐Martinez, J. , Goossens, B. , … Krützen, M. (2017). Morphometric, behavioral, and genomic evidence for a new orangutan species. Current Biology, 27, 3487–3498. 29103940 10.1016/j.cub.2017.09.047

[ajpa24598-bib-0060] Nelson, S. V. (2013). Chimpanzee fauna isotopes provide new interpretations of fossil ape and hominin ecologies. Proceedings of the Royal Society Biological Sciences., 280, 20132324. 24197413 10.1098/rspb.2013.2324 PMC3826229

[ajpa24598-bib-0061] Nowak, M. G. , Rianti, P. , Wich, S. A. , Meijaard, E. , & Fredriksson, G. (2017). *Pongo tapanuliensis*, Tapanuli orangutan. The IUCN Red List of Threatened Species, 8235, e.T120588639A120588662.

[ajpa24598-bib-0062] Oelze, V. M. , Fahy, G. , Hohmann, G. , Robbins, M. M. , Leinert, V. , Lee, K. , Eshuis, H. , Seiler, N. , Wessling, E. G. , Head, J. , Boesch, C. , & Kühl, H. S. (2016). Comparative isotope ecology of African great apes. Journal of Human Evolution, 101, 1–16. 27886808 10.1016/j.jhevol.2016.08.007

[ajpa24598-bib-0063] Oelze, V. M. , Fuller, B. T. , Richards, M. P. , Fruth, B. , Surbeck, M. , Hublin, J.‐J. , & Hohmann, G. (2011). Exploring the contribution and significance of animal protein in the diet of bonobos by stable isotope ratio analysis of hair. Proceedings of the National Academy of Sciences, 108, 9792–9797. 10.1073/pnas.1018502108 PMC3116404 21628564

[ajpa24598-bib-0064] Oelze, V. M. , Head, J. S. , Robbins, M. M. , Richards, M. , & Boesch, C. (2014). Niche differentiation and dietary seasonality among sympatric gorillas and chimpanzees in Loango National Park (Gabon) revealed by stable isotope analysis. Journal of Human Evolution, 66, 95–106. 24373257 10.1016/j.jhevol.2013.10.003

[ajpa24598-bib-0065] Oelze, V. M. , Wittig, R. M. , Lemoine, S. , Kühl, H. S. , & Boesch, C. (2020). How isotopic signatures relate to meat consumption in wild chimpanzees: A critical reference study from Taï National Park, Côte d'Ivoire. Journal of Human Evolution, 146, 102817. 32683168 10.1016/j.jhevol.2020.102817

[ajpa24598-bib-0066] Ogawa, N. , Nagata, T. , Kitazato, H. , & Ohkouchi, N. (2010). Ultra sensitive elemental analyzer/isotope ratio mass spectrometer for stable nitrogen and carbon isotope analyses. In N. Ohkouchi , I. Tayasu , & K. Koba (Eds.), Earth, life, and isotopes (pp. 339–353). Kyoto University Press.

[ajpa24598-bib-0067] O'Leary, M. (1988). Carbon isotopes in photosynthesis. Bioscience, 38, 328–336.

[ajpa24598-bib-0068] Pontzer, H. , Raichlen, D. A. , Shumaker, R. W. , Ocobock, C. , & Wich, S. A. (2010). Metabolic adaptation for low energy throughput in orangutans. Proceedings of the National Academy of Sciences, 107, 14048–14052. 10.1073/pnas.1001031107 PMC2922585 20679208

[ajpa24598-bib-0069] Pushkina, D. , Bocherens, H. , Chaimanee, Y. , & Jaeger, J.‐J. (2010). Stable carbon isotope reconstructions of diet and paleoenvironment from the late middle Pleistocene Snake cave in northeastern Thailand. Die Naturwissenschaften, 97, 299–309. 20127068 10.1007/s00114-009-0642-6

[ajpa24598-bib-0070] Qu, Y. , Jin, C. , Zhang, Y. , Hu, Y. , Shang, X. , & Wang, C. (2014). Preservation assessments and carbon and oxygen isotopes analysis of tooth enamel of *Gigantopithecus blacki* and contemporary animals from Sanhe cave, Chongzuo, South China during the early Pleistocene. Quaternary International, 354, 52–58.

[ajpa24598-bib-0071] R Core Team . (2021). R: A language and environment for statistical computing. R Foundation for Statistical Computing.

[ajpa24598-bib-0072] Reitsema, L. J. (2012). Introducing fecal stable isotope analysis in primate weaning studies. American Journal of Primatology, 74, 926–939. 22729669 10.1002/ajp.22045

[ajpa24598-bib-0073] Reitsema, L. J. , Jones, C. E. , Gilbert, H. R. , Fragaszy, D. , & Izar, P. (2020). Isotopic and elemental corroborates for wild bearded capuchin (*Sapajus libidinosus*) omnivorous dietary adaptation at Fazenda Boa Vista, Brazil. Rapid Communications in Mass Spectrometry, 34, e8856. 32526804 10.1002/rcm.8856

[ajpa24598-bib-0074] Reitsema, L. J. , Partrick, K. A. , & Muir, A. B. (2015). Inter‐individual variation in weaning among rhesus macaques (*Macaca mulatta*): Serum stable isotope indicators of suckling duration and lactation. American Journal of Primatology, 78, 1113–1134. 26284697 10.1002/ajp.22456

[ajpa24598-bib-0075] Roberts, P. , Blumenthal, S. A. , Dittus, W. , Wedage, O. , & Lee‐Thorp, J. A. (2017). Stable carbon, oxygen, and nitrogen, isotope analysis of plants from a south Asian tropical forest: Implications for primatology. American Journal of Primatology, 79, 22656. 10.1002/ajp.22656 28345759

[ajpa24598-bib-0076] Russon, A. E. , Wich, S. A. , Ancrenaz, M. , Kanamori, T. , Knott, C. D. , Kuze, N. , Morrogh‐Bernard, H. C. , Pratje, P. , Ramlee, H. , Rodman, P. , Sawang, A. , Sidiyasa, K. , Singleton, I. , & Van Schaik, C. P. (2009). Geographic variation in orangutan diets. In S. A. Wich , S. S. Utami Atmoko , T. Mitra Setia , & C. P. van Schaik (Eds.), Orangutans: Geographic variation in behavioral ecology and conservation (pp. 135–156). Oxford University Press.

[ajpa24598-bib-0077] Sabah Wildlife Department . (2020). Orangutan action plan for Sabah. Sabah Wildlife Department.

[ajpa24598-bib-0078] Sandberg, P. A. , Loudon, J. E. , & Sponheimer, M. (2012). Stable isotope analysis in primatology: A critical review. American Journal of Primatology, 74, 969–989. 23015270 10.1002/ajp.22053

[ajpa24598-bib-0079] Schoeninger, M. J. (2014). Stable isotope analyses and the evolution of human diets. Annual Review of Anthropology, 43, 413–430.

[ajpa24598-bib-0080] Schoeninger, M. J. , & DeNiro, M. J. (1984). Nitrogen and carbon isotopic composition of bone collagen from marine and terrestrial animals. Geochimica et Cosmochimica Acta, 48, 625–639.

[ajpa24598-bib-0081] Schuppli, C. , Forss, S. I. F. , Meulman, E. J. M. , Zweifel, N. , Lee, K. C. , Rukmana, E. , Vogel, E. R. , van Noordwijk, M. A. , & van Schaik, C. P. (2016). Development of foraging skills in two orangutan populations: Needing to learn or needing to grow? Frontiers in Zoology, 13, 43. 27708679 10.1186/s12983-016-0178-5 PMC5041519

[ajpa24598-bib-0082] Schuppli, C. , Utami Atmoko, S. S. , Vogel, E. R. , van Schaik, C. P. , & van Noordwijk, M. A. (2021). The development and maintenance of sex differences in dietary breadth and complexity in Bornean orangutans. Behavioral Ecology and Sociobiology, 75, 81. 34776592 10.1007/s00265-021-03014-3 PMC8550522

[ajpa24598-bib-0083] Schurr, M. R. , Fuentes, A. , Luecke, E. , Cortes, J. , & Shaw, E. (2012). Intergroup variation in stable isotope ratios reflects anthropogenic impact on the barbary macaques (*Macaca sylvanus*) of Gibraltar. Primates, 53, 31–40. 21881959 10.1007/s10329-011-0268-0

[ajpa24598-bib-0084] Singleton, I. , Wich, S. A. , Nowak, M. , Usher, G. , & Utami‐Atmoko, S. S. (2017). *Pongo abelii*, Sumatran orangutan. The IUCN Red List of Threatened Species, 8235, e.T120588639A120588662.

[ajpa24598-bib-0085] Smith, B. N. , & Epstein, S. (1971). Two categories of ^13^C/^12^C ratios for higher plants. Plant Physiology, 47, 380–384. 16657626 10.1104/pp.47.3.380 PMC365873

[ajpa24598-bib-0086] Smith, T. M. , Austin, C. , Hinde, K. , Vogel, E. R. , & Arora, M. (2017). Cyclical nursing patterns in wild orangutans. Science Advances, 3, e1601517. 28560319 10.1126/sciadv.1601517 PMC5435413

[ajpa24598-bib-0087] Spehar, S. N. , Sheil, D. , Harrison, T. , Louys, J. , Ancrenaz, M. , Marshall, A. J. , Wich, S. A. , Bruford, M. W. , & Meijaard, E. (2018). Orangutans venture out of the rainforest and into the anthropocene. Science Advances, 4, e1701422. 29963619 10.1126/sciadv.1701422 PMC6021148

[ajpa24598-bib-0088] Sponheimer, M. , Codron, D. , Passey, B. H. , de Ruiter, D. J. , Cerling, T. E. , & Lee‐Thorp, J. A. (2009). Using carbon isotopes to track dietary change in modern, historical, and ancient primates. American Journal of Physical Anthropology, 140, 661–670. 19890855 10.1002/ajpa.21111

[ajpa24598-bib-0089] Suess, H. E. (1955). Radiocarbon concentration in modern wood. Science, 122, 415–417.13246648

[ajpa24598-bib-0090] Szpak, P. (2014). Complexities of nitrogen isotope biogeochemistry in plant‐soil systems: Implications for the study of ancient agricultural and animal management practices. Frontiers in Plant Science, 5, 288. 25002865 10.3389/fpls.2014.00288 PMC4066317

[ajpa24598-bib-0091] Tanaka‐Oda, A. , Kenzo, T. , Inoue, Y. , Yano, M. , Koba, K. , & Ichie, T. (2016). Variation in leaf and soil δ^15^N in diverse tree species in a lowland dipterocarp rainforest, Malaysia. Trees ‐ Structure and Function., 30, 509–522.

[ajpa24598-bib-0092] Tayasu, I. , Hirasawa, R. , Ogawa, N. O. , Ohkouchi, N. , & Yamada, K. (2011). New organic reference materials for carbon‐ and nitrogen‐stable isotope ratio measurements provided by Center for Ecological Research, Kyoto University, and Institute of Biogeosciences, Japan Agency for Marine‐Earth Science and Technology. Limnology, 12, 261–266.

[ajpa24598-bib-0093] Tsutaya, T. , Fujimori, Y. , Hayashi, M. , Yoneda, M. , & Miyabe‐Nishiwaki, T. (2017). Carbon and nitrogen stable isotopic offsets between diet and hair/feces in captive chimpanzees. Rapid Communications in Mass Spectrometry, 31, 59–67. 27717069 10.1002/rcm.7760

[ajpa24598-bib-0094] Tsutaya, T. , Mackie, M. , Sawafuji, R. , Miyabe‐Nishiwaki, T. , Olsen, J. V. , & Cappellini, E. (2021). Faecal proteomics as a novel method to study mammalian behaviour and physiology. Molecular Ecology Resources, 21, 1808–1819. 33720532 10.1111/1755-0998.13380 PMC8360081

[ajpa24598-bib-0095] Tsutaya, T. , Ogawa, N. O. , Nomura, T. , Shimizu, M. , Ohkouchi, N. , & Kuze, N. (2021). Carbon and nitrogen stable isotopic offsets between diet and hair/feces in captive orangutans. Primates, 62, 945–954. 34415484 10.1007/s10329-021-00940-8

[ajpa24598-bib-0096] Tsutaya, T. , & Yoneda, M. (2015). Reconstruction of breastfeeding and weaning practices using stable isotope and trace element analyses: A review. Yearbook of Physical Anthropology, 156, 2–21. 10.1002/ajpa.22657 25407359

[ajpa24598-bib-0097] van der Merwe, N. J. , & Medina, E. (1989). Photosynthesis and ^13^C/^12^C ratios in Amazonian rain forests. Geochimica et Cosmochimica Acta, 53, 1091–1094.

[ajpa24598-bib-0098] van der Merwe, N. J. , & Medina, E. (1991). The canopy effect, carbon isotope ratios and foodwebs in Amazonia. Journal of Archaeological Science, 18, 249–259.

[ajpa24598-bib-0099] van Noordwijk, M. A. , Sauren, S. E. B. , Nuzuar, A. A. , Morrogh‐Bernard, H. C. , Utami Atmoko, S. S. , & van Schaick, C. P. (2009). Development of independence Sumatran and Bornean orangutans compared. In Orangutans: Geographic variation in behavioral ecology and conservation (pp. 189–203). Oxford University Press.

[ajpa24598-bib-0100] van Noordwijk, M. A. , & van Schaik, C. P. (2005). Development of ecological competence in Sumatran orangutans. American Journal of Physical Anthropology, 94, 79–94. 10.1002/ajpa.10426 15472890

[ajpa24598-bib-0101] van Noordwijk, M. A. , Willems, E. P. , Utami Atmoko, S. S. , Kuzawa, C. W. , & van Schaik, C. P. (2013). Multi‐year lactation and its consequences in Bornean orangutans (*Pongo pygmaeus wurmbii*). Behavioral Ecology and Sociobiology, 67, 805–814.

[ajpa24598-bib-0102] Vogel, E. R. , Alavi, S. E. , Utami‐Atmoko, S. S. , Van Noordwijk, M. A. , Bransford, T. D. , Erb, W. M. , Zulfa, A. , Sulistyo, F. , Farida, W. R. , & Rothman, J. M. (2017). Nutritional ecology of wild Bornean orangutans (*Pongo pygmaeus wurmbii*) in a peat swamp habitat: Effects of age, sex, and season. American Journal of Primatology, 79, 1–20. 10.1002/ajp.22618 27889926

[ajpa24598-bib-0103] Vogel, E. R. , Crowley, B. E. , Knott, C. D. , Blakely, M. D. , Larsen, M. D. , & Dominy, N. J. (2012). A noninvasive method for estimating nitrogen balance in free‐ranging primates. International Journal of Primatology, 33, 567–587.

[ajpa24598-bib-0104] Vogel, E. R. , Knott, C. D. , Crowley, B. E. , Blakely, M. D. , Larsen, M. D. , & Dominy, N. J. (2012). Bornean orangutans on the brink of protein bankruptcy. Biology Letters, 8, 333–336. 22171019 10.1098/rsbl.2011.1040 PMC3367743

[ajpa24598-bib-0105] White, T. D. , Ambrose, S. H. , Suwa, G. , Su, D. F. , Degusta, D. , Bernor, R. L. , Boisserie, J. R. , Brunet, M. , Delson, E. , Frost, S. , Garcia, N. , Giaourtsakis, L. X. , Haile‐Selassie, Y. , Clark Howell, F. , Lehmann, T. , Likius, A. , Pehlevan, C. , Saegusa, H. , Semprebon, G. , … Vrba, E. (2009). Macrovertebrate paleontology and the Pliocene habitat of *Ardipithecus ramidus* . Science, 326, 87–93. 19810193

[ajpa24598-bib-0106] Wich, S. A. , Gaveau, D. , Abram, N. , Ancrenaz, M. , Baccini, A. , Brend, S. , Curran, L. , Delgado, R. A. , Erman, A. , Fredriksson, G. M. , Goossens, B. , Husson, S. J. , Lackman, I. , Marshall, A. J. , Naomi, A. , Molidena, E. , Nardiyono, Nurcahyo, A. , Odom, K. , Panda, A. , … Meijaard, E. (2012). Understanding the impacts of land‐use policies on a threatened species: Is there a future for the Bornean orang‐utan? PLoS One, 7, e49142. 23145100 10.1371/journal.pone.0049142 PMC3492325

[ajpa24598-bib-0107] Wich, S. A. , Utami‐Atmoko, S. S. , Mitra Setia, T. , Djoyosudharmo, S. , & Geurts, M. L. (2006). Dietary and energetic responses of Pongo abelii to fruit availability fluctuations. International Journal of Primatology, 27, 1535–1550.

[ajpa24598-bib-0108] Wurster, C. M. , Rifai, H. , Zhou, B. , Haig, J. , & Bird, M. I. (2019). Savanna in equatorial Borneo during the late Pleistocene. Scientific Reports, 9, 6392. 31024024 10.1038/s41598-019-42670-4 PMC6483998

