# Supplementary material for: Stable isotopic investigation of the feeding ecology of wild Bornean orangutans
Source: Am J Biol Anthropol. 2022 Aug 10;179(2):276–90. doi: 10.1002/ajpa.24598 (PMC9804541; doi:10.1002/ajpa.24598)
Supplement: Supplementary file 1 — Supplementary Figure S1 Comparison of (a) %C, (b) %N, (c) δ13C, and (d) δ15N values of paired plant samples obtained from the same species + parts and collected during different periods. Supplementary Figure S2. Comparison of plant (a) %C, (b) %N, (c) δ13 C, and (d) δ15N values obtained from different parts of the same species. Supplementary Figure S3. Age change in infant/juvenile–mother difference in (a) %C, (b) %N, (c) δ13 C, and (d) δ15N values of the paired infant/juvenile–mother fecal samples collected within the same month. Raw data were used instead of average data of infant fecal samples collected from the same individual during the same month. Supplementary Table S1. Individual information of the subject orangutans in this study. The estimated age of orangutan individuals, as of September 1st, 2015, with information on the relatively precise birth date. Supplementary Table S4. Summary of the plant samples collected during different periods. Supplementary Table S5. Results of the paired Mann–Whitney U tests for the same plant species + parts collected in different periods. Supplementary Table S6. Explanatory variables and intercept in the linear mixed models for fecal %N of wild orangutans in DVCA. Significant fixed effects are shown in bold. [file AJPA-179-276-s002.pdf]

Supplementary Information

Stable isotopic investigation of feeding ecology of wild Borneo orangutans

Takumi Tsutaya*, Anna Wong, Peter T. Malim, Henry Bernard, Nanako O. Ogawa, Naohiko Ohkouchi, Shun Hongo, Tomoyuki Tajima, Tomoko Kanamori, Noko Kuze

* Corresponding author.

E-mail addresses: tsutaya_takumi@soken.ac.jp, tsutayatakumi@gmail.com (T. Tsutaya).

1. Supplementary Texts

1.1 Exclusion of outliers

Leave-one-out cross-validation was applied to detect outliers in plant and fecal samples. Mean and standard deviation (SD) for every plant species + part were calculated by excluding the target sample. If the sample fulfilled both of the following criteria, it was excluded as an outlier:
i) %C, %N, $\delta^{13}C$, or $\delta^{15}N$ value of the target sample was outside the mean ± 3SD range of the species + part, and ii) Difference in %C, %N, or isotope ratio, ≥6%, ≥3%, or 3‰, respectively, from the average of the species + part. This first screening excluded one plant sample (DF188). Then, mean and SD for all plant samples were calculated by excluding the target sample. If the sample fulfilled both of the following criteria, it was excluded as an outlier: i) %C, %N, $\delta^{13}C$, or $\delta^{15}N$ value of the target sample was outside the mean ± 3SD range of all plant samples, and ii) difference in %C, %N, or isotope ratio, ≥6%, ≥3%, or 3‰, respectively, from an average of all plant samples. Nine plant samples (DF006, DF083, DF093, DF171, DF175, DF178, DF241, DF251, and DF256) were excluded after this second screening.

The mean and SD for all the fecal samples were calculated by excluding the target sample. If the sample fulfilled both of the following criteria, it was excluded as an outlier: i) %C, %N, $\delta^{13}C$, or $\delta^{15}N$ value of the target sample was outside the mean ± 3SD range of the fecal samples, and ii) Difference in %C, %N, or isotope ratio, ≥6%, ≥3%, or 3‰, respectively, from the average of the fecal samples. This screening excluded two fecal samples (DD2016-06 and DD2016-08).

1.2 Comparison with plant stable isotope ratios reported from other Bornean rainforests

Few studies reported isotope ratios of fresh leaves in Bornean rainforests, and most studies measured stable isotope ratios of leaf litter and soil (Pfeiffer et al., 2013; Ridzuan et al., 2017). The mean $\delta^{13}C$ and $\delta^{15}N$ values of leaves obtained from the canopy (n = 11) were −29.7‰ ± 0.5‰ and 0.0‰ ± 0.4‰, respectively; those obtained from understorey (trees at the height of less than 2 m, n = 15) of the mixed lowland dipterocarp forest of Lambir National Park in Sarawak, Borneo, Malaysia, were −33.7‰ ± 0.4‰ and −0.7‰ ± 0.3‰, respectively, (Hyodo et al., 2010). The mean

$\delta^{15}N$ values of leaves obtained from the mixed lowland dipterocarp forest of Lambir showed variation due to the species-specific strategy for nitrogen uptake under low soil nutrient conditions and were approximately 0.3‰ for plant species with ectomycorrhiza root symbiotic microorganisms and −1.6‰ for plant species with arbuscular mycorrhiza root symbiotic microorganisms (Tanaka-Oda et al., 2016). The mean $\delta^{13}C$ and $\delta^{15}N$ values of leaves obtained from broadleaf *Lithocaprus clementianus* were −32.9‰ and −1.7‰, respectively, in the tropical montane forest of Mt. Kinabalu in Sabah, Borneo, Malaysia (Ushio et al., 2017).

The mean $\delta^{13}C$ and $\delta^{15}N$ values (−30.6‰ ± 1.1‰ and 1.1‰ ± 0.8‰, respectively) of orangutans' food leaves in DVCA were similar to those reported for canopy leaves from the mixed lowland dipterocarp forest in Lambir (Hyodo et al., 2010), where the similar forest type was classified. Mann–Whitney U tests showed no significant difference between $\delta^{15}N$ values for leaves obtained in DVCA (0.8‰ ± 1.2‰, $n = 18$) and those reported for plants with symbiotic ectomycorrhizal fungi in Lambir (0.3‰ ± 1.0‰, $n = 22$: Tanaka-Oda et al., 2016) ($U = 245$, $p = 0.206$). However, when compared with $\delta^{15}N$ values for leaves of plants with symbiotic arbuscular mycorrhizal fungi in Lambir (−1.6‰ ± 2.0‰, $n = 47$: Tanaka-Oda et al., 2016), the $\delta^{15}N$ values for leaves obtained in DVCA were significantly greater ($U = 707$, $p < 0.001$).

The $\delta^{15}N$ values for plants in DVCA are a few per mille greater than those reported in Lambir (Hyodo et al., 2012; Tanaka-Oda et al., 2016), which would be a result of the differences in plant species compositions, symbiotic fungi, and the baseline $\delta^{15}N$ values between DVCA and Lambir. Plants show larger variation in $\delta^{15}N$ values with their species-specific strategy for nitrogen uptake (Tanaka-Oda et al., 2016). Therefore, mean $\delta^{15}N$ values of different datasets would be biased if the composition of plant species and symbiont fungi differs. Furthermore, plant $\delta^{15}N$ values vary according to the $\delta^{15}N$ values of the nitrogen source (Evans, 2001; Szpak, 2014).

1.3 Difference in plant stable isotope ratios between two rainfall periods

In most cases, stable isotope ratios of plants collected during lower- or higher-rainfall months (periods 1 and 2, respectively) showed no significant difference. When considering the species + parts collected during different periods, paired Mann–Whitney U tests did not indicate significant differences in %N and $\delta^{15}N$ values but showed significant differences in %C and $\delta^{13}C$ (Supplementary Table S5). The mean %C and $\delta^{13}C$ of plant samples collected during period 1 showed 0.9‰ ± 1.4% increased values and 4.8‰ ± 4.0‰ decreased values compared with those of the paired plant samples with the same species and parts collected during period 2 (Supplementary Table S5; Supplementary Figure S1).

The $\delta^{13}C$ values of plants correlate negatively with mean annual precipitation (Kohn, 2010). But no relationship was observed between plant $\delta^{13}C$ values and rainfall periods in DVCA. This is because i) the seasonal difference in rainfall is relatively small in DVCA, and ii) plants do not

necessarily represent short-term (i.e., several months) changes in precipitation. Although it was not clear why the plant samples collected during period 1 showed increased elemental carbon concentration, there might be a seasonal difference in macronutrient contents due to the different levels of ripening (Conklin-Brittain et al., 1998) or seasonal difference in the composition of plant species that show different carbon contents.

1.4 Elemental N concentrations of feces from flanged male individuals

Adult male includes both flanged and unflanged individuals. For studying the expected difference in the fecal %N of adult flanged males, data points of unflanged adult males (2/16 for averaged and 3/23 for non-averaged data sets) were excluded, and the revised data set was used for the analysis. A linear fixed-effect model considering sex (female or male), age group (infant/juvenile, adolescent, or adult), and sine and cosine of the sampling date within the year as fixed effects and the individual ID (1|individual) as a random effect was constructed for this data set. However, the impact of age group, sex, and interaction could not explain the variation in non-averaged fecal %N (Supplementary Table S6).

References

Conklin-Brittain N Lou, Wrangham RW, Hunt KD. 1998. Dietary response of chimpanzees and cercopithecines to seasonal variation in fruit abundance. II. Macronutrients. Int J Primatol 19:971–998.

Evans RD. 2001. Physiological mechanisms influencing plant nitrogen isotope composition. Trends Plant Sci 6:121–126.

Hyodo F, Matsumoto T, Takematsu Y, Kamoi T, Fukuda D, Nakagawa M, Itioka T. 2010. The structure of a food web in a tropical rain forest in Malaysia based on carbon and nitrogen stable isotope ratios. J Trop Ecol 26:205–214.

Kohn MJ. 2010. Carbon isotope compositions of terrestrial C3 plants as indicators of (paleo)ecology and (paleo)climate. Proc Natl Acad Sci 107:19691–19695.

Pfeiffer M, Mezger D, Dyckmans J. 2014. Trophic ecology of tropical leaf litter ants (Hymenoptera: Formicidae) - A stable isotope study in four types of Bornean rain forest. Myrmecol News 19:31–41.

Ridzuan DS, Rawi CSM, Hamid SA, Al-Shami SA. 2017. Determination of food sources and trophic position in Malaysian tropical highland streams using carbon and nitrogen stable isotopes. Acta Ecol Sin journa 37:97–104.

Szpak P. 2014. Complexities of nitrogen isotope biogeochemistry in plant-soil systems: implications for the study of ancient agricultural and animal management practices. Front Plant Sci 5:288.

Tanaka-Oda A, Kenzo T, Inoue Y, Yano M, Koba K, Ichie T. 2016. Variation in leaf and soil $\delta^{15}N$ in diverse tree species in a lowland dipterocarp rainforest, Malaysia. Trees 30:509–522.

Ushio M, Aiba SI, Takeuchi Y, Iida Y, Matsuoka S, Repin R, Kitayama K. 2017. Plant-soil feedbacks and the dominance of conifers in a tropical montane forest in Borneo. Ecol Monogr 87:105–129.

2. Supplementary Figures

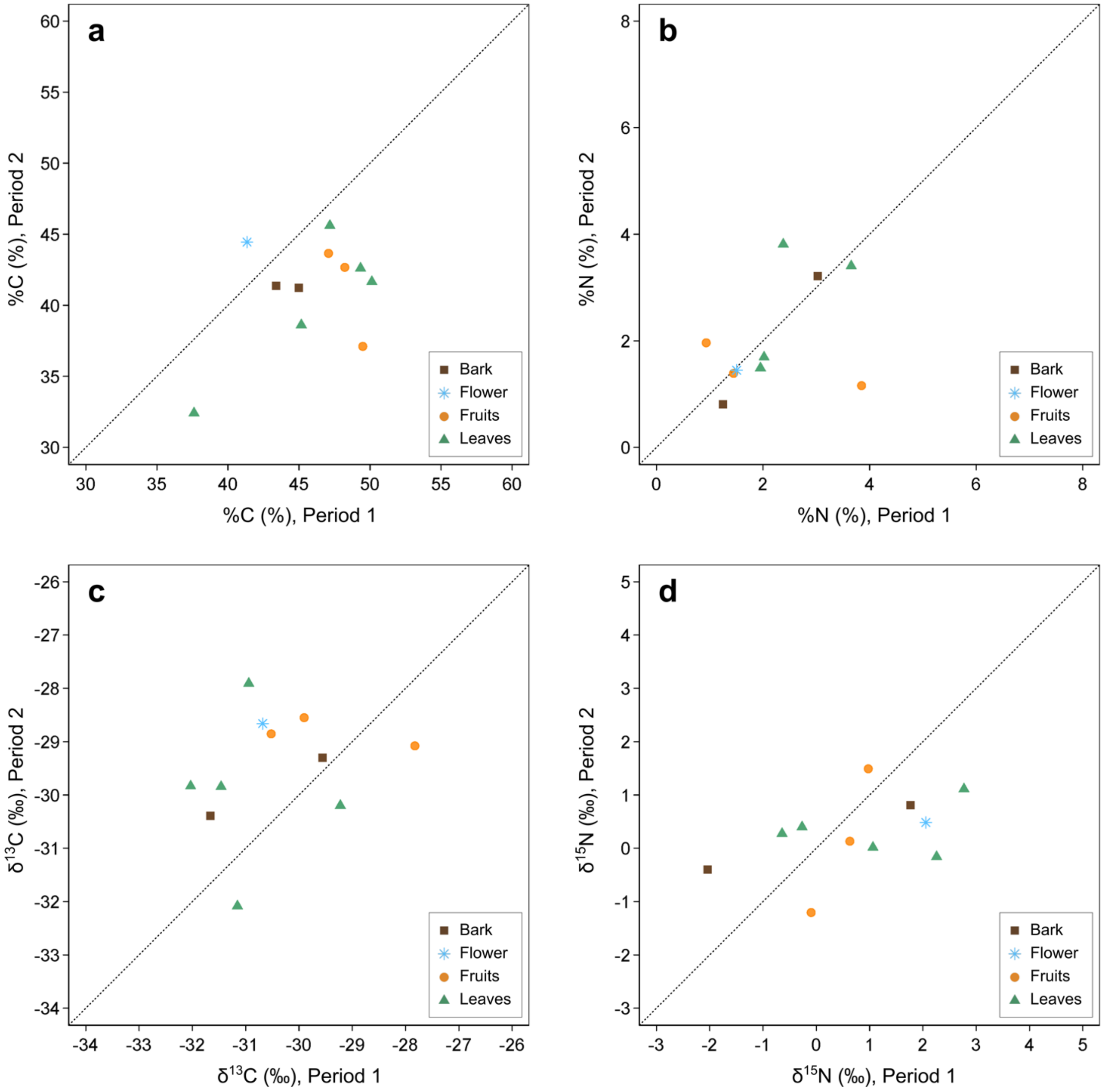

Supplementary Figure S1. Comparison of a) %C, b) %N, c) $\delta^{13}C$, and d) $\delta^{15}N$ values of paired plant samples obtained from the same species + parts and collected during different periods.

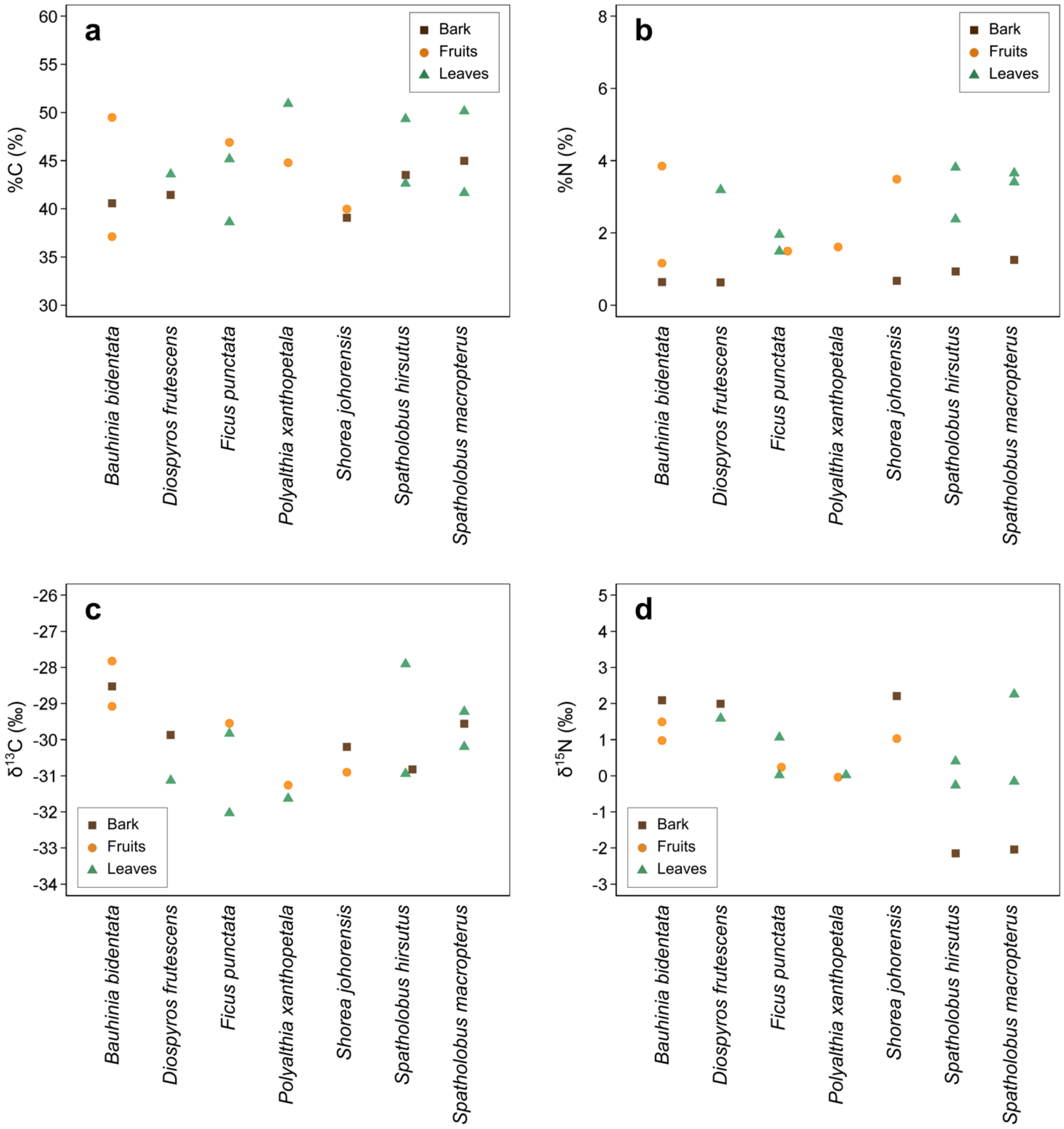

Supplementary Figure S2. Comparison of plant a) %C, b) %N, c) $\delta^{13}C$, and d) $\delta^{15}N$ values obtained from different parts of the same species.

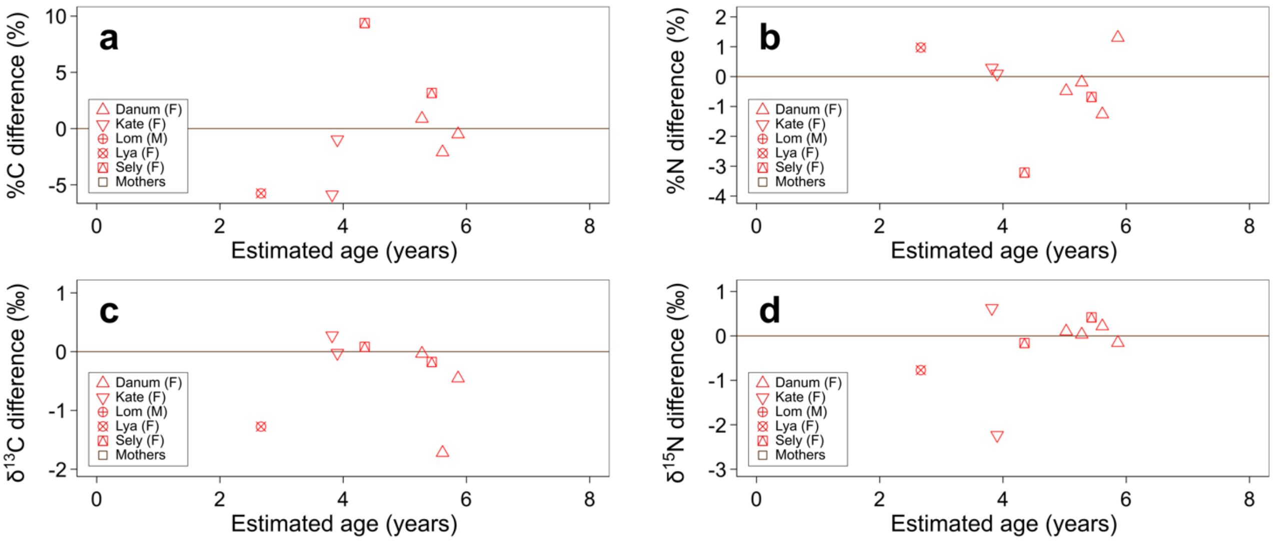

Supplementary Figure S3. Age change in infant/juvenile–mother difference in a) %C, b) %N, c) $\delta^{13}C$, and d) $\delta^{15}N$ values of the paired infant/juvenile–mother fecal samples collected within the same month. Raw data were used instead of average data of infant fecal samples collected from the same individual during the same month.

3. Supplementary Tables

Supplementary Table S1. Individual information of the subject orangutans in this study. The estimated age of orangutan individuals, as of September 1st, 2015, with information on the relatively precise birth date.

Individual	Sex	Age class	Flange status	Maternal status	Estimated age
Abu	Male	Adult	Flanged	–	–
Aco	Male	Adolescent	–	–	10.0
Ali	Male	Adult	Flanged	–	–
Amy	Female	Adolescent	–	–	–
Beth	Female	Adult	–	Mother	–
Danum	Female	Juvenile	–	–	4.5
Gaman	Male	Adult	Flanged	–	–
Gotenz	Male	Adult	Flanged	–	–
Jack	Male	Adult	Unflanged	–	–
Johnny	Male	Adolescent	–	–	–
Kate	Female	Juvenile	–	–	3.1
Khai	Male	Adolescent	–	–	–
Kimi	Male	Adolescent	–	–	6.5
Lina	Female	Adult	–	Mother	–
Linda	Female	Adult	–	Mother	–
Lom	Male	Juvenile	–	–	5.2
Lya	Female	Infant	–	–	1.6
Ruby	Female	Adult	–	–	–
Sely	Female	Juvenile	–	–	4.2
Sheena	Female	Adult	–	Mother	–
Son	Male	Adult	Flanged	–	–
Sumi	Female	Adult	–	Mother	–
Yamato	Female	Adolescent	–	–	–
Yanti	Female	Adult	–	Mother	–

Supplementary Table S2. Carbon and nitrogen stable isotope ratios of the analyzed fecal samples. Excluded samples as an outlier are marked with an asterisk.

[table_s2.xlsx]

Supplementary Table S3. Carbon and nitrogen stable isotope ratios of the analyzed food samples. Orangutan individual that consumed the plant sample was also shown. Excluded samples as an outlier are marked with an asterisk.

[table_s3.xlsx]

Supplementary Table S4. Summary of the plant samples collected during different periods.

Tissue	Period	%C		%N		$\delta^{13}C$		$\delta^{15}N$		n
		Mean	SD	Mean	SD	Mean	SD	Mean	SD	
Bark	1	41.5	3.8	1.7	1.5	-30.3	1.7	0.6	1.8	11
	2	40.6	1.4	1.2	1.0	-30.6	2.0	0.3	1.2	8
Flower	1	41.3	—	1.5	—	-30.7	—	2.1	—	1
	2	44.4	—	1.5	—	-28.7	—	0.5	—	1
Fruits	1	47.0	3.4	1.6	0.7	-30.1	1.5	0.7	1.6	24
	2	41.4	3.0	1.7	0.8	-30.0	1.2	1.1	1.5	8
Leaves	1	45.9	3.9	3.1	1.2	-30.8	0.9	1.0	1.5	10
	2	42.2	5.5	3.0	1.4	-30.4	1.3	0.5	0.7	8
Other	1	40.1	NA	2.6	—	-28.9	—	0.5	—	1
	2	—	—	—	—	—	—	—	—	0

Supplementary Table S5. Results of the paired Mann–Whitney U tests for the same plant species + parts collected in different periods.

	Difference		U-test	
	Mean	SD	U	p-value
%C	0.93	1.44	9	0.032
%N	-0.50	1.27	48	0.206
$\delta^{13}C$	-4.77	4.03	63	0.005
$\delta^{15}N$	-0.16	1.09	35	0.492

Supplementary Table S6. Explanatory variables and intercept in the linear mixed models for fecal %N of wild orangutans in DVCA. Significant fixed effects are shown in bold.

Explanatory variables		Effect	SE	t	p-value
Fixed effect	**Intercept**	**3.46**	**0.43**	**8.05**	**<0.001**
	Sex: Male	0.26	0.56	0.47	0.642
	Age: Adult	-0.20	0.49	-0.41	0.687
	Age: Infant/Juvenile	-0.53	0.56	-0.95	0.357

	Date: Sine	-0.28	0.16	-1.80	0.076
	Date: Cosine	0.33	0.17	1.99	0.051
	Sex: Male × Age: Adult	-0.45	0.67	-0.67	0.511
	Sex: Male × Age: Infant/Juvenile	0.62	0.91	0.68	0.506
Random effect	SD of individual ID	0.13	0.36	–	–
